# Evolution and emergence of primate‐specific interferon regulatory factor 9

**DOI:** 10.1002/jmv.28521

**Published:** 2023-01-31

**Authors:** Sam Drury, Grace Claussen, Allison Zetterman, Hideaki Moriyama, Etsuko N. Moriyama, Luwen Zhang

**Affiliations:** ^1^ School of Biological Sciences University of Nebraska Lincoln Nebraska USA; ^2^ Center for Plant Science Innovation University of Nebraska Lincoln Nebraska USA; ^3^ Nebraska Center for Virology University of Nebraska Lincoln Nebraska USA

**Keywords:** evolution, innate immunity, interferon, IRF, primate

## Abstract

The binding of interferon (IFN) to its receptors leads to formation of IFN‐stimulated gene factor 3 (ISGF3) complex that activates the transcription of cellular IFN‐regulated genes. IFN regulatory factor 9 (IRF9, also called ISGF3γ or p48) is a key component of ISGF3. However, there is limited knowledge regarding the molecular evolution of IRF9 among vertebrates. In this study, we have identified the existence of the *IRF9* gene in cartilaginous fish (sharks). Among primates, several isoforms unique to old world moneys and great apes are identified. These IRF9 isoforms are named as primate‐specific IRF9 (PS‐IRF9) to distinguish from canonical IRF9. PS‐IRF9 originates from a unique exon usage and differential splicing in the *IRF9* gene. Although the N‐terminus are identical for all IRF9s, the C‐terminal regions of the PS‐IRF9 are completely different from canonical IRF9. In humans, two PS‐IRF9s are identified and their RNA transcripts were detected in human primary peripheral blood mononuclear cells. In addition, human PS‐IRF9 proteins were detected in human cell lines. Sharing the N‐terminal exons with the canonical IRF9 proteins, PS‐IRF9 is predicted to bind to the same DNA sequences as the canonical IRF9 proteins. As the C‐terminal regions of IRFs are the determinants of IRF functions, PS‐IRF9 may offer unique biological functions and represent a novel signaling molecule involved in the regulation of the IFN pathway in a primate‐specific manner.

## 1 INTRODUCTION

The innate immune system comprises the cells and the mechanisms that defend the host from infection by other organisms in a nonspecific manner. Type I interferons (IFNs) are the proteins made and released by host cells in response to the presence of pathogens—such as viruses, bacteria, parasites, or tumor cells. IFNs are a key component in the vertebrate innate response to invading pathogens. Once recognizing pathogen‐associated molecular patterns, IFNs will be produced and initiate innate as well as adaptive immune responses against pathogens.^1^, ^2^, ^4^

**Figure 1:**
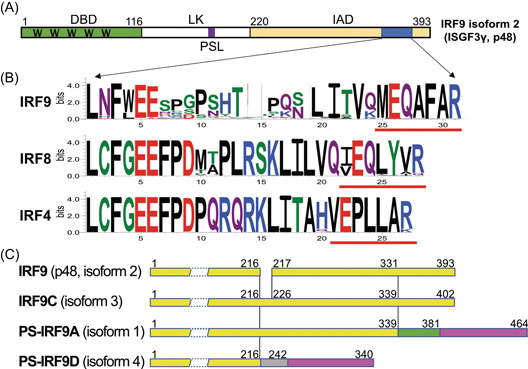
Identification of two unique isoforms/variants of human interferon regulatory factor 9 (IRF9). (A) Domain structures of canonical IRF9 protein. The DNA‐binding domain (DBD), the IRF‐association domain (IAD), the linker region (LK), polyserine linker (PSL), five well‐conserved tryptophans (W) in DBD, and conserved sequences in the C‐terminus in IAD are shown. The amino acid (aa) position numbers are based on the human IRF9 (NP_006075.3). (B) Conserved amino acids found in the C‐terminus of IRF9 proteins in mammals. Sequence logos are used to illustrate the amount of sequence conservation for each position. The overall height of the stack of letters indicates the sequence conservation at each position. The height of symbols within each stack indicates the relative frequency of each amino acid. The multiple sequence alignments were generated using mammalian IRF9, ‐8, and ‐4 protein sequences listed in Supporting Information: Table S1. The illustrated regions correspond to aa 349–376 in the human IRF9 (NP_006075.3), aa 358–385 in the human IRF8 (NP_001350836.1), and aa 445–471 in the human IRF4 (NP_002451.2). The conserved motif sequences for IRF9 and its corresponding sequences in IRF4/8 are underlined. (C) Schematic diagram of human IRF9 proteins. The four isoforms of IRF9 are illustratively compared. Primate‐specific IRF9 (PS‐IRF9A), IRF9C, and PS‐IRF9D sequences are based on NP_001372329.1, NP_001372330.1, and NP_001372331.1, respectively. Solid bars represent protein‐coding region and different colors represent different amino acid sequences. Detailed sequence comparisons are listed in Supporting Information: Figure S2.

IFN regulatory factors (IRFs) are a small family of transcription factors with variety of functions. IRF proteins share extensive similarity in the DNA‐binding domain (DBD) located in the N‐terminus, which is characterized by five well‐conserved tryptophans (Ws in Figure 1A). The DBD region contains a helix‐turn‐helix structure and recognizes a DNA sequence known as IFN‐stimulated response elements (ISRE).^5^ The C‐terminal portion of IRFs contains the IRF‐association domain (IAD), which is variable and defines their specific biological functions (Figure 1A). The IRF family has a variety of functions including, but not limited to, apoptosis, oncogenesis, host defense, and viral latency.^6^, ^7^, ^8^, ^9^, ^10^

IRF9 plays a critical role in Janus kinase–signal transducer and activator of transcription (JAK–STAT) signaling.^11^ IRF9 was first named as IFN‐stimulated gene factor 3γ (ISGF3γ) and p48, because it was first discovered as part of a protein subunit purified from the ISGF3 complex.^12^ As a component of ISGF3, IRF9 mediates the IFN response by binding to ISRE and activating the downstream IFN‐stimulated genes (ISGs).^13^ Other than as a subunit in the ISGF3, IRF9 also regulates many other cellular processes that are related to the pathogenesis of diseases including cancer, cardiovascular, and inflammatory diseases.^14^, ^15^, ^16^, ^17^, ^18^, ^19^

Albeit exciting findings have been made since its discovery, molecular evolutionary studies of IRF9 have been limited.^20^, ^21^, ^22^, ^23^ Therefore, in this study, we searched IRF9 proteins from a wide range of vertebrates and compared their sequences. We have established that cartilaginous fish (sharks) are the first group of organisms that possesses IRF9 during vertebrate evolution. In addition, we have identified IRF9 isoforms unique to primate lineages and named them as primate‐specific IRF9 (PS‐IRF9). As PS‐IRF9 is associated with primate evolution, they may play a unique role in a variety of the primate‐specific functions related to IFNs and beyond.

## 2 RESULTS

### 2.1 IRF9 first emerged in cartilaginous fish

**Figure 2:**
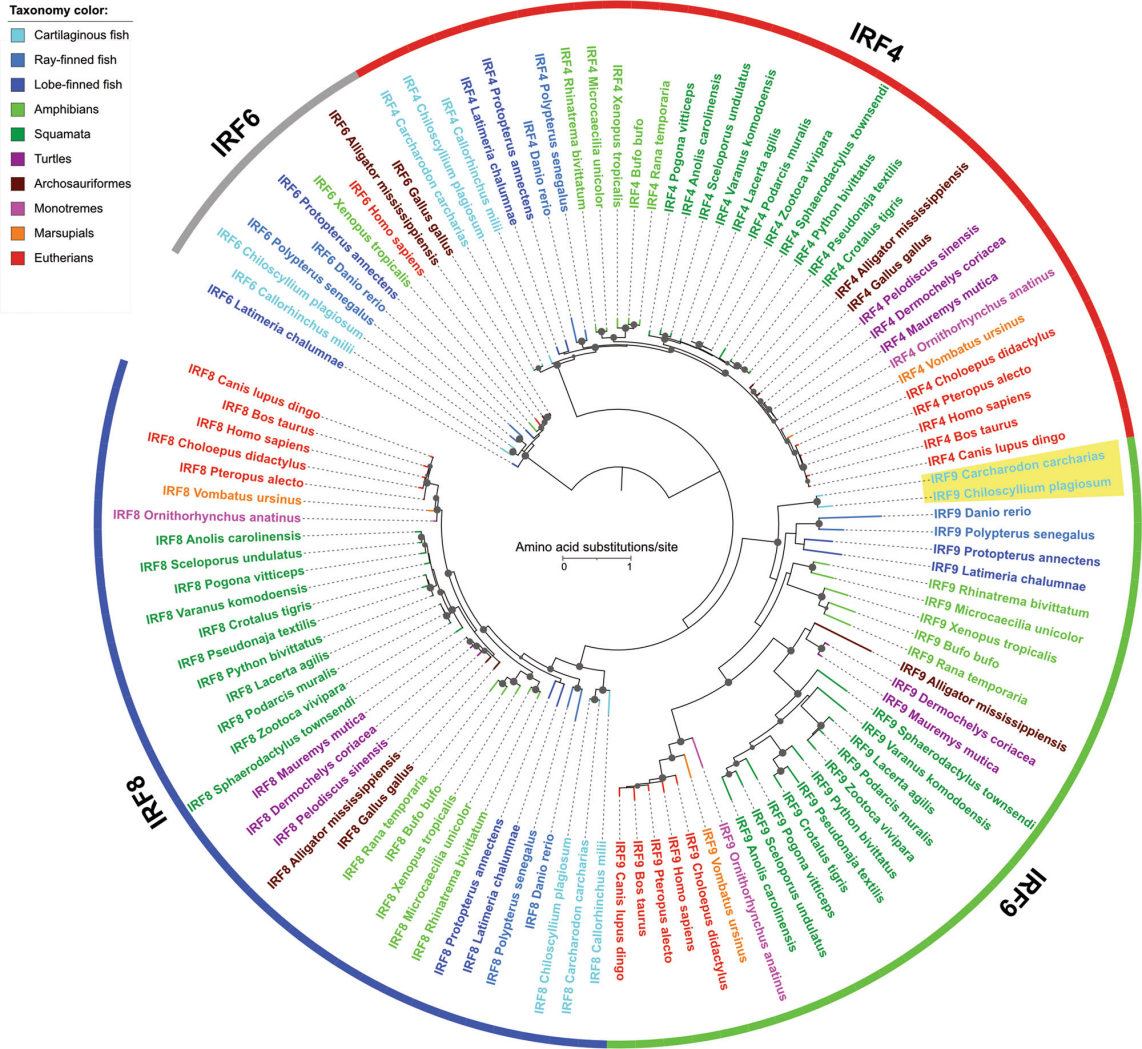
Phylogenetic relationships among interferon regulatory factors IRF4, IRF8, and IRF9 protein families. The maximum likelihood phylogeny was reconstructed using the IRF9 protein sequences from 32 species and those of IRF8 and IRF4 from 35 species. The IRF6 protein sequences from 10 representative species were also included as the outgroup. The internal nodes supported by 80% or higher by both ultrabootstrap and SH‐aLRT branch test are denoted by gray dots. The size of the dots is correlated with the ultrabootstrap values (80%–100%). External branches and sequence names are colored based on the taxonomic groups as shown in the color legend. The newly identified shark IRF9s are indicated with the yellow highlight. See Supporting Information: Tables S1, S2, and S3 for the protein sequences used.

Due to their rapid evolution, it is difficult to trace the origin and evolutionary history of IFN sequences. As IRF9 is a critical gene involved for IFN signaling, studying the evolution of IRF9 would shed a light on the molecular evolutionary process of the IFN system. Therefore, we conducted systematic searches of IRF9 and related IRF protein sequences, and identified their IRF memberships based on their similarities and phylogenetic locations (Supporting Information: Tables S1 and S2). It has been thought that during the vertebrate evolution, *IRF9* genes first appeared in bony fish (Osteichthyes), one of the two major clades of the jawed vertebrates (Gnathostomata). However, our phylogenetic analysis among IRF proteins clearly indicated IRF9 is present in cartilaginous fishes (sharks; Chondrichthyes), although they have been annotated as “IRF8‐like” in the public databases (Figure 2 and Supporting Information: Table S2). IRF9 has not been identified in Cephalochordata (such as lancelets) nor in Urochordata (tunicates) and Agnatha (lampreys).^21^, ^22^, ^24^ Therefore, IRF9 likely first emerged in cartilaginous fishes, which coincided with the emergence of a primitive Type I IFN system.^25^

We also identified IRF9 proteins from lobe‐finned fishes (lungfish and coelacanth; Sarcopterygii). Similar to what was observed for shark IRF9s, these proteins are misannotated as “IRF8‐like” in the database (Supporting Information: Table S2). However, based on the phylogenetic analysis, it is evident that after its emergence in ancestral bony vertebrates, IRF9 proteins have been maintained during the entire vertebrate evolution, although they have become more divergent than other IRF proteins (Figure 2).

### 2.2 Identification of a conserved motif unique to IRF9 proteins in mammals

Previously, we compared the DBD regions among nine IRF families and identified that PYKVY as a signature sequence for the IRF4/8/9 subfamily.^26^ By comparing the IAD protein sequences within the IRF4/8/9 subfamily in mammals, the conserved signature motif for IRF9 (MEQAFAR) in placental mammals is identified (Figure 1B and Supporting Information: Figure S1 and Table S1).

**Figure 3:**
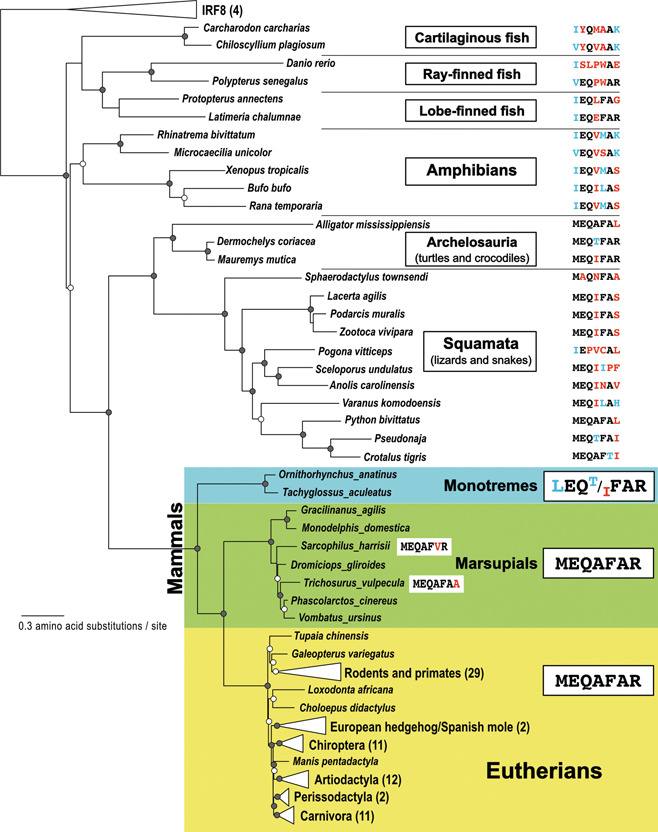
Distribution of the MEQAFAR motifs in the vertebrate evolution. The maximum‐likelihood phylogenies of interferon regulatory factor 9 (IRF9) proteins were reconstructed from 103 IRF9 as well as 4 IRF8 protein sequences as the outgroup (see Supporting Information: Tables S1 and S2 for the sequences used). Internal nodes supported by 70% or higher by both or either of ultrabootstrap and SH‐aLRT branch test are denoted by closed or open circles. For each species or group of organisms, the amino acid sequence corresponding to the MEQAFAR motif is shown on the right. When the amino acid is different from the corresponding amino acid in MEQAFAR, it is shown in either blue (for similar amino acids) or red (for dissimilar amino acids).

By mapping this IRF9‐specific conserved motif sequence on the phylogeny, the evolution of IRF9 proteins can be studied more in detail. Primitive forms of IRF9s are first emerged in the sharks, where the motif regions are occupied by quite different amino acids (Figure 3). By the time of the emergence of reptiles, gradually, the sequences in the motif region have evolved into those similar to the current motif (MEQAFAR). Mammals are divided into two major groups: Monotremata and Theria. The platypus (*Ornithorhynchus anatinus*) and soft‐beaked echidna (*Tachyglossus aculeatus*) are extant species of monotremes, the only mammals that lay eggs instead of giving birth to live young. Monotremes have the LEQ(T/I)FAR sequences. The phylogenetic distribution of the motif sequences indicates that the first amino acid of the motif was changed from “M” to “L” in the monotreme lineage (Figure 3 and Supporting Information: Table S1). Therian mammals include marsupials and placental mammals. In the marsupial lineage, the MEGAFAR motif sequence has been largely conserved. Among placental mammals, the conserved MEGAFAR sequences are present in almost all of IRF9 proteins examined (Figure 3 and Supporting Information: Table S1). Clearly, the conserved motif, MEQAFAR, is favored through mammalian evolution. As the IAD region of IRF9 proteins, especially in the C‐terminus, are generally more divergent, it suggests that the important role of the motif in IRF9 protein functions and it must have been under strong functional constraints.

### 2.3 Identification of PS‐IRF9 in primates

When IRF9 protein sequences in primates were examined, we had found a portion of IRF9 isoforms do not contain the MEGAFAR motif. In humans, four entries are listed for proteins and transcripts of human IRF9 on the National Center for Biotechnology Information Reference Sequences (NCBI RefSeqs) (GeneID: 10379). The Isoform 2 is the canonical IRF9 (p48 or ISGF3γ; NM_006084.5 and NP_006075.3). The protein sequence of the Isoform 3 is identical to the Isoform 2, except nine amino acid deletion. However, the other two IRF9 isoform sequences (Isoforms 1 and 4) have large differences from the sequence of the canonical IRF9. Although all IRF9 isoforms have common sequences in the N‐terminus, the IRF9 Isoforms 1 and 4 have completely different C‐terminal sequences (indicated in different colors in Figure 1C). This is caused by alternative splicing generating frame‐shifts of the open reading frames (ORFs) that produce completely different amino acid sequences at the C‐terminal region (Supporting Information: Figure S2).

**Figure 4:**
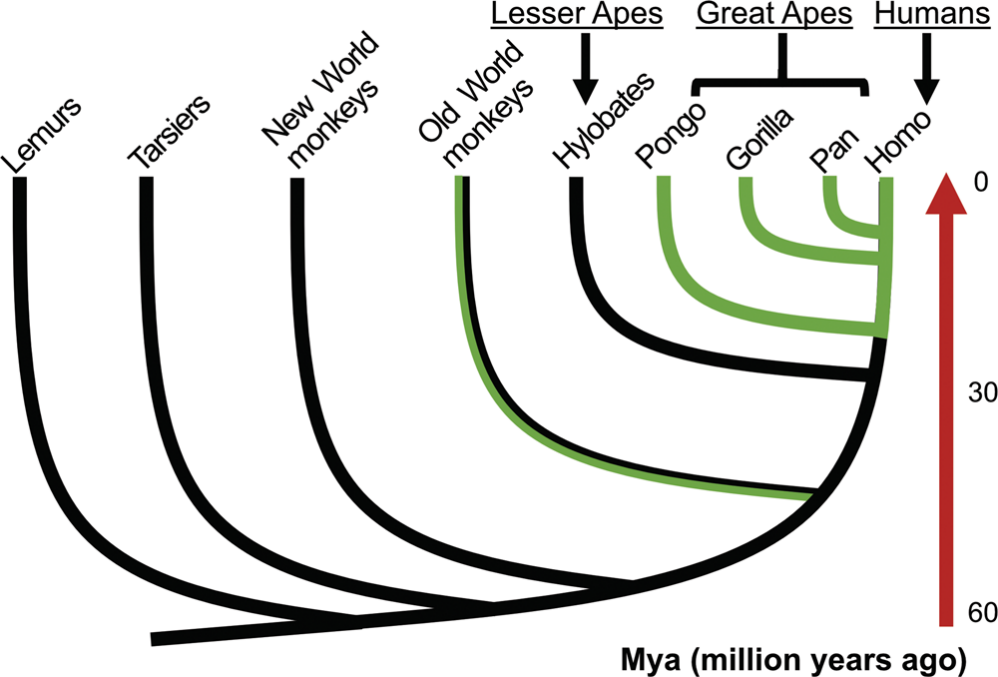
The emergence of primate‐specific interferon regulatory factor 9 (PS‐IRF9) during the primate evolution. Evolutionary relationship among the primate species used in this study is illustrated. Canonical IRF9 is found in all of these primate species. Green lines indicate on which lineages the acquisition of PS‐IRF9 occurred. Not all old world monkeys have PS‐IRF9 and mixed colored lines indicate the facts. Approximated phylogenetic relationships and age estimates for diversifications among primates are based on refs.^27^, ^28^, ^29^, ^30^, ^31^ Numbers on the right represent approximate divergence time in million years ago (Mya).

In addition to humans, similar IRF9 isoforms are identified in great apes as well as in old world monkeys based on current sequence deposits in the databases (Figure 4, Table 1, and Supporting Information Figure S3 and Table S4). We therefore have named this new class of IRF9 as PS‐IRF9, because they are found only in the primate lineages. In humans, the four protein isoforms are named as IRF9 (canonical IRF9, Isoform 2), IRF9C (Isoform 3), PS‐IRF9A (Isoform 1), and PS‐IRF9D (Isoform 4) (Figure 1C). Of note, PS‐IRF9D is apparently only present in humans based on currently available sequence data. Gorillas (as well as Colobus) do not have the similar sequences as human PS‐IRF9s. Instead, it has two unique splicing variants with different amino acid sequences (Supporting Information: Figure S4 and Table 1). The gorilla IRF9 protein sequences are also primate‐specific based on the current sequence deposits. In addition, we tried to trace the earliest form of the PS‐IRF9 and identified in three species that have short sequences similar to PS‐IRF9 (Table 1 and Supporting Information: Figure S5).

**Table 1:** Distribution of PS‐IRF9 in various species.

Species	Common name	# of protein isoforms	# of canonical IRF9	# of PS‐ IRF9	% of PS‐IRF9
*Homo sapiens*	Human	4	2	2	50
*Pan paniscus*	Bonobo	5	2	3	60
*Pan troglodytes*	Chimpanzee	5	2	3	60
*Gorilla gorilla gorilla* a	Western lowland gorilla	4	2	2	50
*Pongo abelii*	Sumatran orangutan	2	1	1	50
*Trachypithecus francoisi*	Francois' leaf monkey	3	1	2	67
*Papio anubis*	Olive baboon	5	4	1	20
*Macaca mulatta*	Rhesus macaque	2	1	1	50
*Chlorocebus sabaeus*	Green monkey	2	1	1	50
*Colobus angolensis palliates* a	Peters' Angolan colobus	2	1	1	50
*Mustela ermine* ^b^	Stoat	3	2	1	33
*Mustela putorius furo* ^b^	Domestic ferret	2	1	1	50
*Lontra canadensis* ^b^	North America river otter	2	1	1	50
*Bos taurus*	Cattle	3	3	0	0
*Mus musculus*	Mouse	3	3	0	0
*Monodelphis domestica*	Gray short‐tailed opossum	4	4	0	0
*Orcinus orca*	Killer whale	2	2	0	0

Abbreviations: IRF9, interferon regulatory factor 9; PS‐IRF9, primate‐specific interferon regulatory factor 9.

^a^ The species have primate‐specific IRF9 but with C‐terminal sequences different from human PS‐IRF9 (Supporting Information: Figure S4).

^b^ The species have PS‐IRF9‐like gene sequences with only a stretch of sequence similarities (Supporting Information: Figure S5).

**Figure 5:**
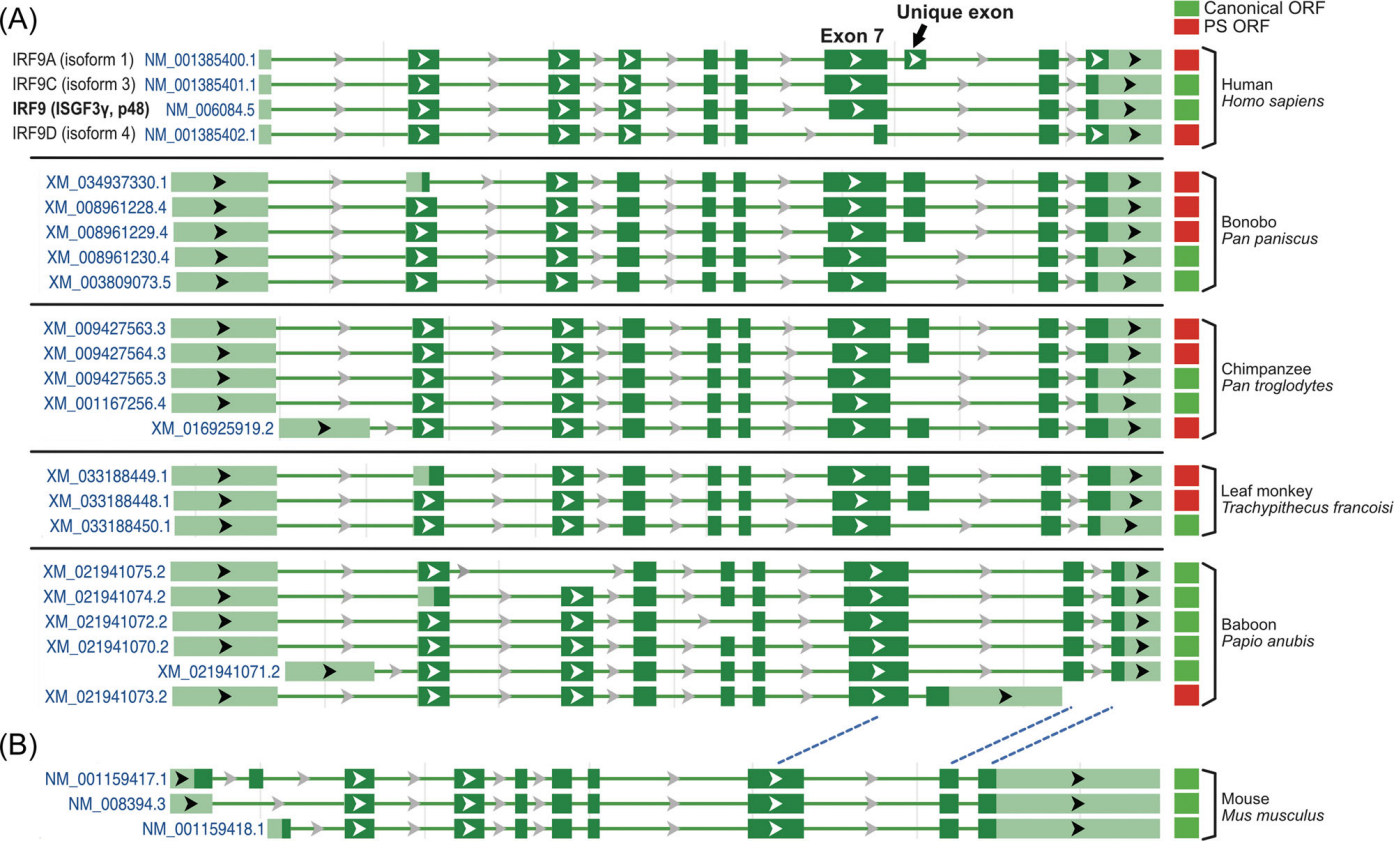
Structure of interferon regulatory factor 9 (IRF9) transcripts. (A) Primate IRF9 transcripts. *IRF9* gene structures from human (gene ID: 10379), bonobo (100973587), chimpanzee (744767), baboon (101020807), and Francois' leaf monkey (117069921) are as shown. Light green bars represent exon sequences and dark green bars represent open reading frames (ORFs). The canonical IRF9 ORFs are indicated with green solid squares and primate‐specific IRF9 (PS‐IRF9) ORFs are indicated with solid red square on the right. The accession numbers are listed on the left. The species are listed on the right. The diagrams are directly obtained from the NCBI gene report pages. The scales of the diagrams have been adjusted for comparison purpose among species. (B) Mouse IRF9 transcripts. The mouse (Gene ID:16391) *IRF9* gene structure is as shown. The potential exons corresponding to primate IRF9 exons are indicated by dashed blue lines.

To address the biogenesis of PS‐IRF9, we examined the relationship among RNA splicing and coding sequence usages in *IRF9* genes. As shown in Figure 5, there is a unique exon (corresponding to the green sequences in Supporting Information: Figure S2) used by humans and other primates for PS‐IRF9A. Rodents, for example, do not use this exon and do not have PS‐IRF9 isoform (Figure 5B). These data collectedly suggest that PS‐IRF9 must have offered some advantageous traits, especially in primates and must have been kept during their evolution.

### 2.4 PS‐IRF9s are present in human cells

**Figure 6:**
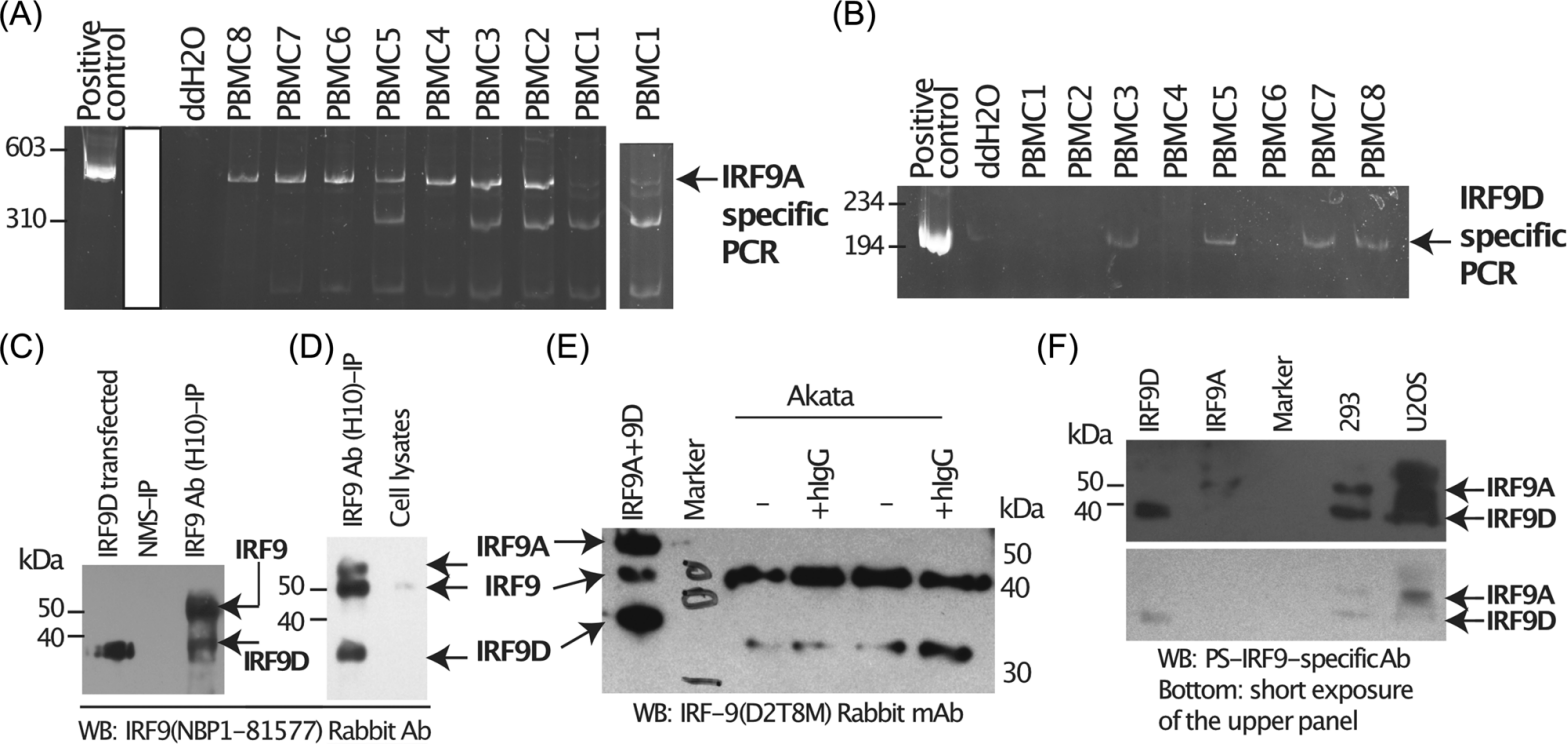
Detection of primate‐specific interferon regulatory factor 9 (PS‐IRF9) in human cells. (A, B) Total RNAs were isolated from eight healthy individuals' peripheral blood mononuclear cells (PBMCs) and used for reverse‐transcription polymerase chain reaction (RT‐PCR) analysis with specific primers for PS‐IRF9A (A) and PS‐IRF9D (B). The PCR products were analyzed in an 8% polyacrylamide gel. The second lane for PBMC1 in A is the overexposure of the same lane on the left. The plasmids for IRF9, PS‐IRF9A, and 9D are mixed at 1:1:1 ratio and used as positive controls. Numbers on the left indicate DNA size makers (in bp). (C) HEK293 cell lysates were immunoprecipitated (IP) with mouse monoclonal anti‐ISGF‐3γ p48 antibody (H‐10) (Santa Cruz, # sc‐365893) or normal mouse serum (NMS). The immunoprecipitates were separated in a 10% sodium dodecyl‐sulfate polyacrylamide gel electrophoresis (SDS‐PAGE). Western blotting (WB) with rabbit IRF9 Ab (Novus Biologicals, NBP1‐81577) was conducted. The transfected PS‐IRF9D lysates were used as a control. (D) HEK293 cells were treated with IFN‐β (100 IU/ml) overnight, the cell lysates were used for IP‐WB as in the C. (E) Cell lysates from Akata cells were used for WB analysis with IRF9 (D2T8M) rabbit monoclonal antibody (mAb) (Cell Signaling, #76684). Akata cells were treated with human IgG (hIgG) for induction of Epstein–Barr virus (EBV) lytic replication. HEK294 cells were transfected with canonical IRF9A and 9D, respectively, and their lysates were mixed at 1:1 ratio and used as a positive control for WBs. Molecular weight markers are shown on the left for C and D, and on the right for E. All antibodies used here are against common regions of all IRF9s. (F) HEK294 cells were transfected with IRF9A and 9D plasmid, respectively, and their lysates were used as a positive control. Cells lysates from HEK293 and U2OS were separated in a 12% SDS‐PAGE. WB with rabbit PS‐IRF9‐specific Ab was conducted. The identities of the signals are as shown.

We examined whether PS‐IRF9 is present in humans at both RNA and protein levels. A common primer for all IRF9 transcripts as well as primers specific to IRF9A or 9D were synthesized, respectively. Total RNAs from eight healthy individuals' peripheral blood mononuclear cells (PBMCs) were used for reverse‐transcription polymerase chain reaction (RT‐PCR) analysis. All eight samples have IRF9A transcripts and 50% of them (four samples) contained IRF9D transcripts (Figures 6A,B). We did detect some additional bands in the samples, which were not present in the positive controls. The identities of those additional bands are currently unknown.

Because of decent differences in the number of amino acids among IRF9 isoforms (Figure 1C), an antibody against the N‐terminal IRF9 may be able to detect the various forms of IRF9 based on their molecular weights. HEK293 cell lysates were first immunoprecipitated with IRF9 mouse monoclonal antibody, then the immunoprecipitation products were used for Western blot analyses with rabbit IRF9 antibody. A band, similar to overexpressed IRF9D, was observed (Figure 6C). IRF9 per se is an ISG. Therefore, we reasoned that PS‐IRF9s might be also ISGs. When HEK293 cells were treated with Type I IFNs, two specific bands, in addition to the one for the canonical IRF9, were detected (Figure 6D). The sizes were predicted to be consistent to those for PS‐IRF9A and 9D. In addition, one IRF9 antibody (Cell Signaling, D2T8M) could detect endogenous IRF9 in a reasonably consistent manner. Akata cells are an Epstein‐Barr virus (EBV)‐positive Burkitts' lymphoma line and human IgG treatment can induce virus replications in Akata. A specific band (molecular weight was similar to IRF9D) was detected (Figure 6E). Finally, an antibody specifically against a peptide sequence unique to PS‐IRF9, but not to the canonical IRF9, was generated. The antibody recognized two bands, corresponding to the PS‐IRF9s, in human cell lines (Figure 6F). These results show that PS‐IRF9s are present in human cells in both RNA and protein forms.

## 3 DISCUSSION

### 3.1 Evolution of IRF9

IRF9 was initially discovered as a component of the potent transcription factor ISGF3 responsible in initiating transcription of hundreds of ISGs to mount antiviral response, which is further implicated in expansive roles across various fields such as cancer.^32^, ^33^, ^34^ Therefore, the IRF9 has to adapt for two aspects: its own biological functions and virus–host interactions.

In this study, we have identified two shark IRFs as the most primitive IRF9 based on phylogenic analyses (Figure 2). Previously, we have identified that a PYKVY motif sequences are present in the DBD region of the IRF4/8/9 subfamily proteins.^26^ Now we have identified uniquely conserved sequences in the IAD region of mammalian IRF9s (Figure 1B). The MEGAFAR or similar sequences were identified from reptiles, monotremes, and marsupials. The MEGAFAR motif is strongly conserved in placental mammals, indicating an importance of this motif sequence in the IRF9 function in this group of organisms (Figure 3).

The amino acid positions 217–377 of the canonical IRF9, the region containing MEQAFAR, are required for ISGF3 complex formation.^35^, ^36^, ^37^ It, therefore, suggests that the motif may be important for the ISGF3 formation and thereby IFN signaling. Interestingly, a structural analysis based on the published IRF9‐STAT2 complex^38^ indicates that the MEQAFAR motif region does not involve in the direct interactions with STAT2 (data not shown). The exact function of the MEQAFAR motif in IRF9 needs further investigation.

Emergence of IRF9 coincided with the formation of the primitive Type I IFN system.^25^ Although this strongly suggested the important functional role of IRF9 in IFN signaling, an alternative factor for IRF9 might be present in some species. (1) Although only a few divergent exceptions were identified (e.g., XP_033928400.1, XP_031363717, and TRZ10743.1), bird genomes usually lack *IRF9* genes.^21^ (2) IRF9 is not found in many fish genomes. (3) Variations found in the MEQAFAR motif per se (Figure 3) suggests a flexibility in their structures for potential ISGF3 complex formations. Therefore, it is quite interesting to examine whether some other IRFs or IRF‐like molecules have similar functions as IRF9 for IFN signaling.

### 3.2 PS‐IRF9 molecule may have additional functions

In this report, we have identified a specific class of IRF9 isoforms in primates and confirmed their presence in human cells (Figure 6 and Table 1). Unique exon usages in primates have generated those PS‐IRF9s (Figure 5). Although a trace of PS‐IRF9‐like sequences was detected in mustelidae members (Table 1 and Supporting Information: Figure S5), PS‐IRF9 are found only in primates, especially in old word monkeys and great apes including humans (Figure 4).

We are in the process to explore the functions of PS‐IRF9. In humans, the amino acid positions 217–377 of the canonical IRF9 are required for ISGF3 complex formation.^35^, ^36^, ^37^ PS‐IRF9s lack this C‐terminal region partially (Isoform 1) or entirely (Isoform 4). Both PS‐IRF9s, without the conserved MEQAFAR in the C‐terminal region, is likely not able to form the ISGF3 complex. However, IRF9 and PS‐IRF9 have the identical N‐terminal DBD region and the intact polyserine linker (PSL) (Figure 1A,C). The PSL is considered as a flexible, disordered spacer that enhances domain interactions and substrate accessibility,^39^ which may be used to keep both the N‐terminus and C‐terminus domain intact. Therefore, the PS‐IRF9 is predicted to bind to the same DNA sequences as the canonical IRF9 and likely to act as a negative regulator for IFN signaling by direct DNA‐binding competitions to the same sites with the canonical IRF9. PS‐IRF9 may be used for fine‐tuning of the IFN pathways in primates. Although no significant domains nor motifs were identified in the C‐terminus of PS‐IRF9, because C‐terminal regions are the determinants of IRF functions, the unique C‐terminus may offer PS‐IRF9 unique biological functions. Understanding the functions of PS‐IRF9 may not only contribute to our knowledge about IFN pathway, but also help exploring the species variations of innate immunity and IRF biology at the molecular levels.

## 4 MATERIALS AND METHODS

### 4.1 Searching of IRF proteins

The sequences of the human IRF protein were used as the queries (see Supporting Information: Tables S1, S2, and S3 for accession numbers) to perform protein similarity searches using BLASTP against the nonredundant protein database at NCBI with the default options. Sequences were collected from mammals, reptiles, birds, amphibians, and fishes. When more than one isoforms were available from the same species, one isoform that was most similar to those from other species was selected. Protein sequences that were partial and too short were excluded. All sequences collected in this study are listed in Supporting Information: Tables S1, S2, and S3.

### 4.2 Phylogenetic analysis of IRF proteins

The grouping of IRF proteins was confirmed using phylogenetic analysis. The protein sequences were aligned using MAFFT v7 with the E‐INS‐i iterative refinement method. The maximum likelihood phylogeny was reconstructed using IQ‐Tree release 1.6.11 with the default options and the amino acid substitution model estimated. Branch support values were obtained using the ultrafast bootstrap support and SH‐aLRT branch test both with 1000 replicates. Representative IRF6 protein sequences were included as the outgroup (Supporting Information: Table S3).

Starting with the IRF protein sequences obtained from the BLASTP searches, alignment and phylogenetic analysis were iteratively performed. After identical sequences were removed and grouping of IRF4, IRF6, IRF8, and IRF9 protein families was established, the alignment was performed again using each IRF family. The final alignment including all IRF sequences was performed using protein sequences from each of the IRF‐family specific alignment as the profile and using the “merge” alignment of MAFFT with the E‐INS‐i iterative refinement method. The visualization of the phylogenies was performed using Interactive Tree of Life website.^40^ All data sets including multiple sequence alignments are available upon request.

### 4.3 Sequence logos

A sequence logo was generated from the protein sequence alignment using WebLogo v3 (https://weblogo.threeplusone.com/create.cgi). For simplicity, the composition adjustment was suppressed. The amino acid “chemistry” color scheme was chosen.

### 4.4 Cells and plasmids

Human primary PBMCs from healthy individuals were gifts from Dr. Wei Jiang. Human embryonic kidney fibroblast 293 (HEK293, CRL‐1573) was obtained from the American Type Culture Collection. The U2OS osteosarcoma cell line was a gift from Dr. Aiming Peng. These cells were grown and maintained in Dulbecco's modified Eagle's medium containing 10% fetal bovine serum (FBS) and 1× penicillin‐streptomycin (PS) in a humidified chamber with 5% CO_2_ at 37°C. Akata cells are an EBV‐positive Burkitt's lymphoma cell line and EBV lytic replication can be induced by the treatments of human IgG (hIgG). Akata cells were cultured in RPMI medium with 10% FBS and 1% PS at 37°C. PS‐IRF9A and 9D expression plasmids were synthesized and cloned into pcDNA3 expression vector by Synbio Technologies, LLC.

### 4.5 RT‐PCR

A common primer (5′‐GGATCAGAGGTCCCTGGAG‐3′) for all IRF9 transcripts as well as IRF9A (5′‐GATATGCAACACACAAGCGCAG‐3′) or 9D (5′‐GAGCCATGGCTCTCTTCCC‐3′) specific primers are made by Thermo Fisher Scientific Inc. Total RNAs were isolated from eight healthy individuals' PBMCs with Trizol method (Invitrogen™ TRIzol™ Reagent, Thermo Fisher Scientific Inc.). Invitrogen™ SuperScript™ First‐Strand Synthesis System was used for complementary DNA synthesis and RT‐PCR analyses. The common primer plus IRF9A‐specific one generated a 461 bp DNA and plus IRF9D‐specific one generated a 202 bp product.

### 4.6 Antibody production and western blot analysis with enhanced chemiluminescence (ECL)

Rabbit polyclonal antibody against IRF9A peptide (amino acids 432–446 in IRF9A), “ADSRAAGSHSVPGVE‐C,” was custom made by GenScript. The last cysteine was added to link the peptide to KLH for antibody production. Anti‐ISGF‐3γ p48 mouse monoclonal antibody (H‐10) and goat anti‐mouse IgG‐HRP (sc‐2005) were purchased from Santa Cruz (sc‐365893). Normal mouse serum was made in our laboratory from C57BL/6 mice. The rabbit IRF9 Abs were from Novus Biologicals (NBP1‐81577) and Cell Signaling (D2T8M, #76684). Goat‐anti‐Rabbit IgG‐HRP (#7074) were purchased from Cell Signaling.

Separation of proteins on sodium dodecyl‐sulfate polyacrylamide gel electrophoresis was carried out following the standard protocol. After the proteins were transferred to a nitrocellulose or Immobilon membrane, the membrane was blocked with 5% nonfat dry milk in TBST (50 mm Tris‐HCl pH 7.5, 200 mm NaCl, 0.05% Tween‐20) at room temperature for 30 min. It was washed briefly with TBST and incubated with the primary antibody in 1% milk in TBST for 1 h at room temperature, or overnight at 4°C. After washing the membrane with TBST three times (10 min each), it was incubated with the secondary antibody at room temperature for 1 h. The membrane was then washed three times with TBST, treated with ECL detection reagents, and exposed to BlueBlot™ HS film from Life Science Products (XR‐0810‐100).

## AUTHOR CONTRIBUTIONS

Sam Drury, Grace Claussen, Allison Zetterman, and Hideaki Moriyama collected and analyzed the data. Luwen Zhang and Etsuko N. Moriyama conceived the idea, analyzed the data, and wrote the manuscript.

## CONFLICT OF INTEREST STATEMENT

The authors declare no conflict of interest.

## Supporting information

Supporting information.

## Data Availability

The data that support the findings of this study are available in the supplementary material of this article.

## References

[1] Ivashkiv LB , Donlin LT . Regulation of type I interferon responses. Nat Rev Immunol. 2014;14:36‐49. 24362405 10.1038/nri3581 PMC4084561

[2] McNab F , Mayer‐Barber K , Sher A , Wack A , O'Garra A . Type I interferons in infectious disease. Nat Rev Immunol. 2015;15:87‐103. 25614319 10.1038/nri3787 PMC7162685

[3] Samuel CE . Antiviral actions of interferons. Clin Microbiol Rev. 2001;14:778‐809. 11585785 10.1128/CMR.14.4.778-809.2001 PMC89003

[4] Sen GC . Viruses and interferons. Annu Rev Microbiol. 2001;55:255‐281. 11544356 10.1146/annurev.micro.55.1.255

[5] Darnell JE, Jr , Kerr M , Stark GR . Jak‐STAT pathways and transcriptional activation in response to IFNs and other extracellular signaling proteins. Science. 1994;264:1415‐1421. 8197455 10.1126/science.8197455

[6] Honda K , Takaoka A , Taniguchi T . Type I interferon [corrected] gene induction by the interferon regulatory factor family of transcription factors. Immunity. 2006b;25:349‐360. 16979567 10.1016/j.immuni.2006.08.009

[7] Honda K , Yanai H , Negishi H , et al. IRF‐7 is the master regulator of type‐I interferon‐dependent immune responses. Nature. 2005;434:772‐777. 15800576 10.1038/nature03464

[8] Tamura T , Yanai H , Savitsky D , Taniguchi T . The IRF family transcription factors in immunity and oncogenesis. Annu Rev Immunol. 2008;26:535‐584. 18303999 10.1146/annurev.immunol.26.021607.090400

[9] Zhang L , Pagano JS . Interferon regulatory factor 7: a key cellular mediator of LMP‐1 in EBV latency and transformation. Sem Cancer Biol. 2001;11:445‐453. 10.1006/scbi.2001.0411 11669606

[10] Zhang L , Pagano JS . Review: structure and function of IRF‐7. J Interferon Cytokine Res. 2002;22:95‐101. 11846980 10.1089/107999002753452700

[11] Stark GR , Darnell JE, Jr . The JAK‐STAT pathway at twenty. Immunity. 2012;36:503‐514. 22520844 10.1016/j.immuni.2012.03.013 PMC3909993

[12] Fu XY , Kessler DS , Veals SA , Levy DE , Darnell JE, Jr . ISGF3, the transcriptional activator induced by interferon alpha, consists of multiple interacting polypeptide chains. Proc Natl Acad Sci USA. 1990;87:8555‐8559. 2236065 10.1073/pnas.87.21.8555 PMC54995

[13] Honda K , Takaoka A , Taniguchi T . Type I inteferon gene induction by the interferon regulatory factor family of transcription factors. Immunity. 2006a;25:349‐360. 16979567 10.1016/j.immuni.2006.08.009

[14] Huber M , Suprunenko T , Ashhurst T , et al. IRF9 prevents CD8(+) T cell exhaustion in an extrinsic manner during acute lymphocytic choriomeningitis virus infection. J Virol. 2017;91:e01219‐17. 28878077 10.1128/JVI.01219-17 PMC5660491

[15] Jiang DS , Luo YX , Zhang R , et al. Interferon regulatory factor 9 protects against cardiac hypertrophy by targeting myocardin. Hypertension. 2014;63:119‐127. 24144649 10.1161/HYPERTENSIONAHA.113.02083

[16] Luker KE , Pica CM , Schreiber RD , Piwnica‐Worms D . Overexpression of IRF9 confers resistance to antimicrotubule agents in breast cancer cells. Cancer Res. 2001;61:6540‐6547. 11522652

[17] Nan J , Wang Y , Yang J , Stark GR . IRF9 and unphosphorylated STAT2 cooperate with NF‐κB to drive IL6 expression. Proc Natl Acad Sci USA. 2018;115:3906‐3911. 29581268 10.1073/pnas.1714102115 PMC5899435

[18] Paul A , Tang TH , Ng SK . Interferon regulatory factor 9 structure and regulation. Front Immunol. 2018;9:1831. 30147694 10.3389/fimmu.2018.01831 PMC6095977

[19] Smith S , Fernando T , Wu PW , et al. MicroRNA‐302d targets IRF9 to regulate the IFN‐induced gene expression in SLE. J Autoimmun. 2017;79:105‐111. 28318807 10.1016/j.jaut.2017.03.003

[20] Du K , Zhong Z , Fang C , et al. Ancient duplications and functional divergence in the interferon regulatory factors of vertebrates provide insights into the evolution of vertebrate immune systems. Dev Comp Immunol. 2018;81:324‐333. 29253557 10.1016/j.dci.2017.12.016

[21] Huang B , Qi ZT , Xu Z , Nie P . Global characterization of interferon regulatory factor (IRF) genes in vertebrates: glimpse of the diversification in evolution. BMC Immunol. 2010;11:22. 20444275 10.1186/1471-2172-11-22 PMC2885996

[22] Nehyba J , Hrdlickova R , Bose HR . Dynamic evolution of immune system regulators: the history of the interferon regulatory factor family. Mol Biol Evol. 2009;26:2539‐2550. 19638535 10.1093/molbev/msp167 PMC2767096

[23] Qi D , Chao Y , Liang J , et al. Adaptive evolution of interferon regulatory factors is not correlated with body scale reduction or loss in schizothoracine fish. Fish Shellfish Immunol. 2018;73:145‐151. 29246809 10.1016/j.fsi.2017.12.013

[24] Kasamatsu J , Oshiumi H , Matsumoto M , Kasahara M , Seya T . Phylogenetic and expression analysis of lamprey toll‐like receptors. Dev Comp Immunol. 2010;34:855‐865. 20363250 10.1016/j.dci.2010.03.004

[25] Secombes CJ , Zou J . Evolution of interferons and interferon receptors. Front Immunol. 2017;8:209. 28303139 10.3389/fimmu.2017.00209 PMC5332411

[26] Angeletti M , Hsu WLN , Majo N , Moriyama H , Moriyama EN , Zhang L . Adaptations of interferon regulatory factor 3 with transition from terrestrial to aquatic life. Sci Rep. 2020;10:4508. 32161340 10.1038/s41598-020-61365-9 PMC7066157

[27] Chapman CA , Lambert JE . Habitat alteration and the conservation of African primates: case study of Kibale National Park, Uganda. Am J Primatol. 2000;50:169‐185. 10711532 10.1002/(SICI)1098-2345(200003)50:3<169::AID-AJP1>3.0.CO;2-P

[28] Lucas PW , Dominy NJ , Riba‐Hernandez P , et al. Evolution and function of routine trichromatic vision in primates. Evolution. 2003;57:2636‐2643. 14686538 10.1111/j.0014-3820.2003.tb01506.x

[29] Pääbo S . The mosaic that is our genome. Nature. 2003;421:409‐412. 12540910 10.1038/nature01400

[30] Page SL , Chiu C , Goodman M . Molecular phylogeny of old world monkeys (Cercopithecidae) as inferred from γ‐Globin DNA sequences. Mol Phylogenet Evol. 1999;13:348‐359. 10603263 10.1006/mpev.1999.0653

[31] Pecon‐Slattery J . Recent advances in primate phylogenomics. Annu Rev Anim Biosci. 2014;2:41‐63. 25384134 10.1146/annurev-animal-022513-114217

[32] Kolosenko I , Fryknäs M , Forsberg S , et al. Cell crowding induces interferon regulatory factor 9, which confers resistance to chemotherapeutic drugs. Int J Cancer. 2015;136:E51‐E61. 25156627 10.1002/ijc.29161

[33] Qian X , Tan H , Zhang J , et al. Identification of biomarkers for pseudo and true progression of GBM based on radiogenomics study. Oncotarget. 2016;7:55377‐55394. 27421136 10.18632/oncotarget.10553 PMC5342424

[34] Weihua X , Lindner DJ , Kalvakolanu DV . The interferon‐inducible murine p48 (ISGF3γ) gene is regulated by protooncogene c‐myc. Proc Natl Acad Sci. 1997;94:7227‐7232. 9207073 10.1073/pnas.94.14.7227 PMC23799

[35] Horvath CM , Stark GR , Kerr IM , Darnell JE, Jr . Interactions between STAT and non‐STAT proteins in the interferon‐stimulated gene factor 3 transcription complex. Mol Cell Biol. 1996;16:6957‐6964. 8943351 10.1128/mcb.16.12.6957 PMC231699

[36] Martinez‐Moczygemba M , Gutch MJ , French DL , Reich NC . Distinct STAT structure promotes interaction of STAT2 with the p48 subunit of the Interferon‐α‐stimulated transcription factor ISGF3. J Biol Chem. 1997;272:20070‐20076. 9242679 10.1074/jbc.272.32.20070

[37] Veals SA , Santa Maria T , Levy DE , Veals SA , Santa Maria T . Two domains of ISGF3 gamma that mediate protein‐DNA and protein‐protein interactions during transcription factor assembly contribute to DNA‐binding specificity. Mol Cell Biol. 1993;13:196‐206. 8417326 10.1128/mcb.13.1.196-206.1993 PMC358899

[38] Rengachari S , Groiss S , Devos JM , Caron E , Grandvaux N , Panne D . Structural basis of STAT2 recognition by IRF9 reveals molecular insights into ISGF3 function. Proc Natl Acad Sci USA. 2018;115:E601‐E609. 29317535 10.1073/pnas.1718426115 PMC5789952

[39] Howard MB , Ekborg NA , Taylor LE , Hutcheson SW , Weiner RM . Identification and analysis of polyserine linker domains in prokaryotic proteins with emphasis on the marine bacterium microbulbifer degradans. Prot Sci. 2004;13:1422‐1425. 10.1110/ps.03511604 PMC2286767 15075401

[40] Letunic I , Bork P . Interactive tree of life (iTOL) v5: an online tool for phylogenetic tree display and annotation. Nucleic Acids Res. 2021;49:W293‐W296. 33885785 10.1093/nar/gkab301 PMC8265157

