# Supplementary material for: Evolution and emergence of primate‐specific interferon regulatory factor 9
Source: J Med Virol. 2023 Jan 31;95(2):e28521. doi: 10.1002/jmv.28521 (PMC10107944; doi:10.1002/jmv.28521)
Supplement: Supplementary file 1 — Supporting information. [file JMV-95-0-s001.pdf]

Supplemental Figure S1: Sequence conservation in the C-terminal regions of IRF9 (A), IRF8 (B), and IRF4 (C). Sequence logos are used to illustrate the amount of sequence conservation for each position. The overall height of the stack of letters indicates the sequence conservation at each position. The height of symbols within each stack indicates the relative frequency of each amino acid. The multiple sequence alignments were generated using mammalian IRF9, -4, and -8 protein sequences listed in Supplementary Table S1. Only the relevant sequences at the C-terminal regions are shown.

Supplemental Figure S2: Protein sequence comparison among the four human IRF9 isoforms. Panel A: Comparison of human IRF9 isoform protein sequences. Sequences are based on NP_006075.3 (IRF9, isoform 2), NP_001372330.1 (IRF9C, isoform 3), NP_001372329.1 (IRF9A, isoform 1), and NP_001372331.1 (IRF9D, isoform 4). The position numbers shown at the top are based on the canonical IRF9 (isoform 2) sequence. The positions shown with cyan background correspond to exon boundaries. For IRF9A and IRF9D (isoforms 1 and 4), alternative splicing caused frame-shifts producing completely different protein sequences at the C-terminal region (shown in lower cases). The MEAQFAR motif is underlined. The sequences are colored similarly to what shown in Figure 1C. The frame-shifting details of the boxed region are explained in **B**. **Panel B: Frame-shifting relationships among human IRF9 isoforms.** The position numbers shown on the left are based on the isoform 1 mRNA sequence (NM_001385400.1). The first codon corresponds to the amino acid position 292 in **A**. Amino acid sequences translated in three frames are aligned below the nucleotide sequence. Amino acid sequences are colored consistent to those shown in **A**. The amino acid sequence derived from the first frame corresponds to the IRF9 isoform 1 product (IRF9A). For isoforms 2 and 3, only

sequences with yellow background are used with the second half using the second frame. For isoform 4, the frame shifts from the first (yellow background) to the third (gray background) then again to the first (magenta background). The MEAQFAR motif is indicated with underline.

Supplemental Figure S3: **Alignment of the PS-IRF9 proteins among primates.** PS-IRF9 sequences from various species are aligned. The green underlines indicate the sequences are similar to the canonical IRF9, and the red lines represent the unique sequences of PS-IRF9A.

Supplemental Figure S4: Protein sequence comparison among IRF9A sequences in human, gorilla, and colobus. PS-IRF9A sequences from human (NP_001372329.1), gorilla (XP_018864869.1 and XP_018864868.1), and Peter's Angolan colobus (XP_011786161.1) were aligned by Clustal Omega. Green lines represent the regions similar to the canonical IRF9 sequences, and the red lines represent PS-IRF9A specific sequences.

Supplemental Figure S5: Alignment of human PS-IRF9A sequences with those of mustelidae family members. PS-IRF9A sequences from Stoat (XP_032199789.1), river otter (XP_044923785.1), ferret (XP_032712190.1), and human (NP_001372329.1) were aligned by Clustal Omega. Green lines represent the regions similar to the canonical IRF9 sequences, and the red lines represent PS-IRF9A specific sequences.

Figure S1

A: IRF9 B: IRF8 C: IRF4

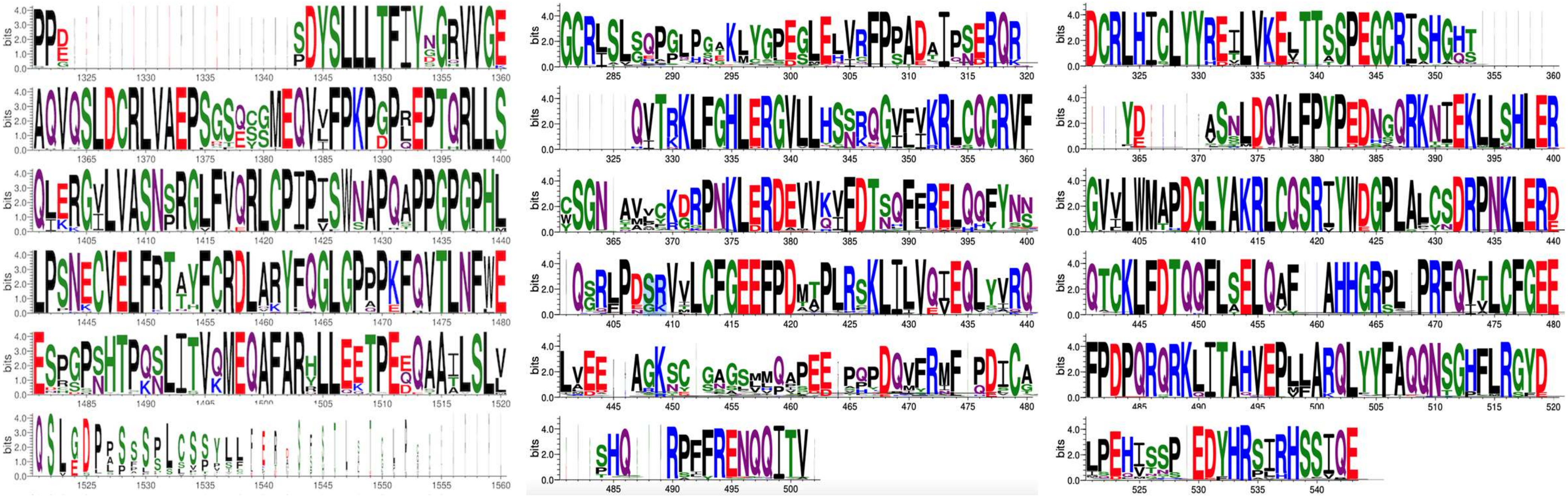

Figure S2

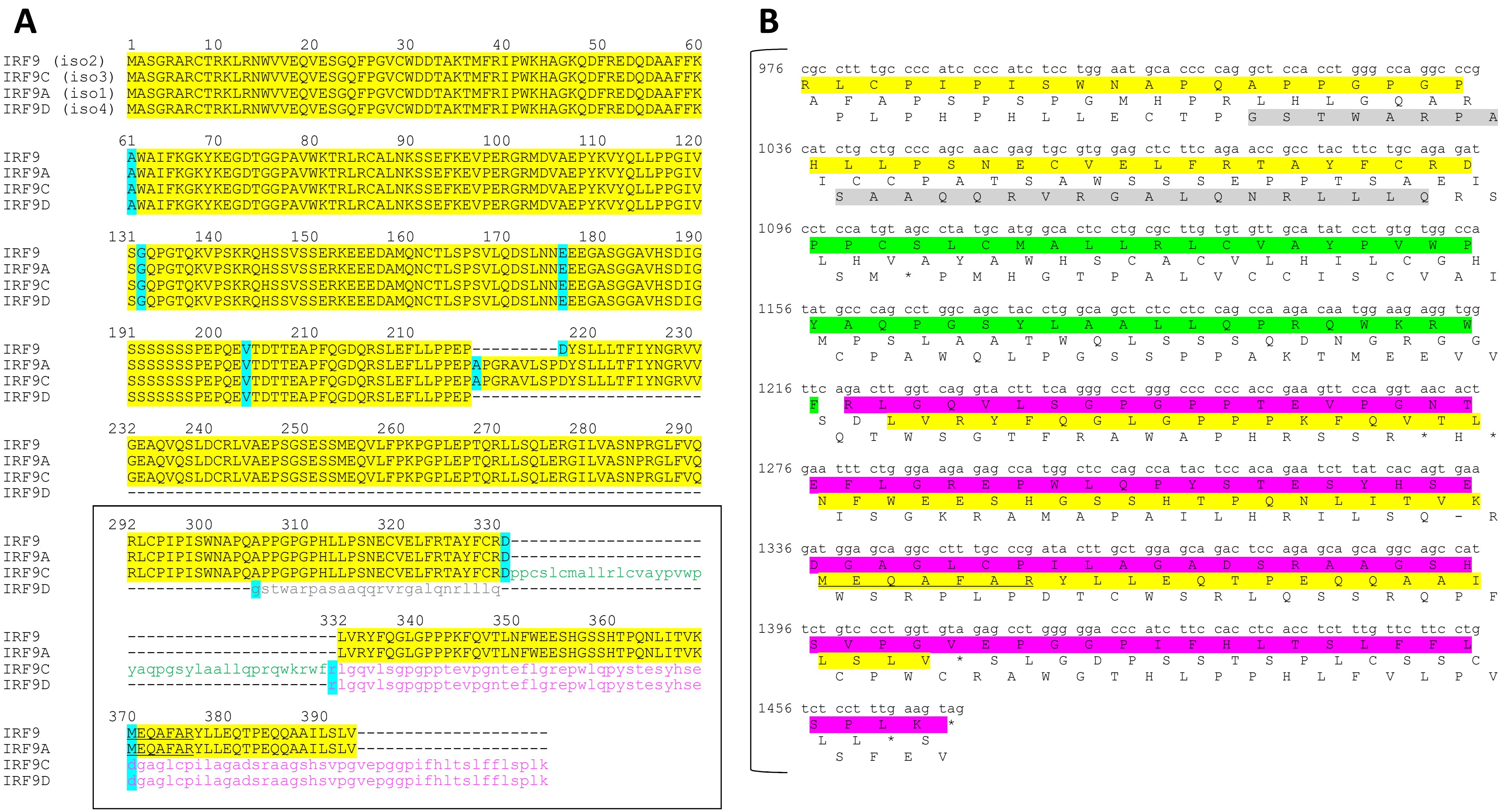

A
1 10 20 30 40 50 60
IRF9 (iso2) MASGRARCTRKLRNWVVEQVESGQFPGVCWDDTAKTMFRIPWKHAGKQDFREDQDAAFFK
IRF9C (iso3) MASGRARCTRKLRNWVVEQVESGQFPGVCWDDTAKTMFRIPWKHAGKQDFREDQDAAFFK
IRF9A (iso1) MASGRARCTRKLRNWVVEQVESGQFPGVCWDDTAKTMFRIPWKHAGKQDFREDQDAAFFK
IRF9D (iso4) MASGRARCTRKLRNWVVEQVESGQFPGVCWDDTAKTMFRIPWKHAGKQDFREDQDAAFFK
61 70 80 90 100 110 120
IRF9 AWAIFKGKYKEGDTGGPAVWKTRLRCALNKSSEFKEVPERGRMDVAEPYKVYQLLPPGIV
IRF9A AWAIFKGKYKEGDTGGPAVWKTRLRCALNKSSEFKEVPERGRMDVAEPYKVYQLLPPGIV
IRF9C AWAIFKGKYKEGDTGGPAVWKTRLRCALNKSSEFKEVPERGRMDVAEPYKVYQLLPPGIV
IRF9D AWAIFKGKYKEGDTGGPAVWKTRLRCALNKSSEFKEVPERGRMDVAEPYKVYQLLPPGIV
131 140 150 160 170 180 190
IRF9 SGQPGTQKVPSKRQHSSVSSERKEEEDAMQNCTLSPSVLQDSLNNEEEGASGGAVHSDIG
IRF9A SGQPGTQKVPSKRQHSSVSSERKEEEDAMQNCTLSPSVLQDSLNNEEEGASGGAVHSDIG
IRF9C SGQPGTQKVPSKRQHSSVSSERKEEEDAMQNCTLSPSVLQDSLNNEEEGASGGAVHSDIG
IRF9D SGQPGTQKVPSKRQHSSVSSERKEEEDAMQNCTLSPSVLQDSLNNEEEGASGGAVHSDIG
191 200 210 210 220 230
IRF9 SSSSSSSPEPQEVTDTTEAPFQGDQRSLEFLLPPEP----------DYSLLLTFIYNGRVV
IRF9A SSSSSSSPEPQEVTDTTEAPFQGDQRSLEFLLPPEPAPGRAVLSPDYSLLLTFIYNGRVV
IRF9C SSSSSSSPEPQEVTDTTEAPFQGDQRSLEFLLPPEPAPGRAVLSPDYSLLLTFIYNGRVV
IRF9D SSSSSSSPEPQEVTDTTEAPFQGDQRSLEFLLPPEP------------------------
232 240 250 260 270 280 290
IRF9 GEAQVQSLDCRLVAEPSGSESSMEQVLFPKPGPLEPTQRLLSQLERGILVASNPRGLFVQ
IRF9A GEAQVQSLDCRLVAEPSGSESSMEQVLFPKPGPLEPTQRLLSQLERGILVASNPRGLFVQ
IRF9C GEAQVQSLDCRLVAEPSGSESSMEQVLFPKPGPLEPTQRLLSQLERGILVASNPRGLFVQ
IRF9D --
292 300 310 320 330
IRF9 RLCPIPISWNAPQAPPGPGPHLLPSNECVELFRTAYFCRD---------------------
IRF9A RLCPIPISWNAPQAPPGPGPHLLPSNECVELFRTAYFCRD---------------------
IRF9C RLCPIPISWNAPQAPPGPGPHLLPSNECVELFRTAYFCRDppcslcmallrlcvaypvwp
IRF9D -------------gstwarpasaaqqrvrgalqnrlllq----------------------
332 340 350 360
IRF9 ----------------------LVRYFQGLGPPPKFQVTLNFWEESHGSSHTPQNLITVK
IRF9A ----------------------LVRYFQGLGPPPKFQVTLNFWEESHGSSHTPQNLITVK
IRF9C yaqpgsylaallqprqwkrwfrlgqvlsgpgpptevpgnteflgrepwlqpystesyhse
IRF9D ---------------------rlgqvlsgpgpptevpgnteflgrepwlqpystesyhse
370 380 390
IRF9 MEQAFARYLLEQTPEQQAAILSLV--------------------
IRF9A MEQAFARYLLEQTPEQQAAILSLV--------------------
IRF9C dgaglcpilagadsraagshsvpgvepggpifhltslfflsplk
IRF9D dgaglcpilagadsraagshsvpgvepggpifhltslfflsplk
B
976 cgc ctt tgc ccc atc ccc atc tcc tgg aat gca ccc cag gct cca cct ggg cca ggc ccg
R L C P I P I S W N A P Q A P P G P G P
A F A P S P S P G M H P R L H L G Q A R
P L P H P H L L E C T P G S T W A R P A
1036 cat ctg ctg ccc agc aac gag tgc gtg gag ctc ttc aga acc gcc tac ttc tgc aga gat
H L L P S N E C V E L F R T A Y F C R D
I C C P A T S A W S S S E P P T S A E I
S A A Q Q R V R G A L Q N R L L L Q R S
1096 cct cca tgt agc cta tgc atg gca ctc ctg cgc ttg tgt gtt gca tat cct gtg tgg cca
P P C S L C M A L L R L C V A Y P V W P
L H V A Y A W H S C A C V L H I L C G H
S M * P M H G T P A L V C C I S C V A I
1156 tat gcc cag cct ggc agc tac ctg gca gct ctc ctc cag cca aga caa tgg aag agg tgg
Y A Q P G S Y L A A L L Q P R Q W K R W
M P S L A A T W Q L S S S Q D N G R G G
C P A W Q L P G S S P P A K T M E E V V
1216 ttc aga ctt ggt cag gta ctt tca ggg cct ggg ccc ccc acc gaa gtt cca ggt aac act
F R L G Q V L S G P G P P T E V P G N T
S D L V R Y F Q G L G P P P K F Q V T L
Q T W S G T F R A W A P H R S S R * H *
1276 gaa ttt ctg gga aga gag cca tgg ctc cag cca tac tcc aca gaa tct tat cac agt gaa
E F L G R E P W L Q P Y S T E S Y H S E
N F W E E S H G S S H T P Q N L I T V K
I S G K R A M A P A I L H R I L S Q - R
1336 gat gga gca ggc ctt tgc ccg ata ctt gct gga gca gac tcc aga gca gca ggc agc cat
D G A G L C P I L A G A D S R A A G S H
M E Q A F A R Y L L E Q T P E Q Q A A I
W S R P L P D T C W S R L Q S S R Q P F
1396 tct gtc cct ggt gta gag cct ggg gga ccc atc ttc cac ctc acc tct ttg ttc ttc ctg
S V P G V E P G G P I F H L T S L F F L
L S L V * S L G D P S S T S P L C S S C
C P W C R A W G T H L P P H L F V L P V
1456 tct cct ttg aag tag
S P L K *
L L * S
S F E V

Supplemental Figure S3: Alignment of the PS-IRF9 among primates.

```
Chimpanzee      MASGRARCTRKLRNWVVEQVESGQFPGVCWDDTAKTMFRIPWKHAGKQDFREDQDAAFFK    60
Orangutan       MASGRARCTRKLRNWVVEQVESGQFPGVCWDDTAKTMFRIPWKHAGKQDFREDQDAAFFK    60
Bonobo          MASGRARCTRKLRNWVVEQVESGQFPGVCWDDTAKTMFRIPWKHAGKQDFREDQDAAFFK    60
Human           MASGRARCTRKLRNWVVEQVESGQFPGVCWDDTAKTMFRIPWKHAGKQDFREDQDAAFFK    60
Leaf monkey     MASGRARCTRKLRNWVVEQVESGQFPGVCWDDTAKTMFRIPWKHAGKQDFREDQDAAFFK    60
Macaque         MASGRARCTRKLRNWVVEQVESGQFPGVCWDDTAKTMFRIPWKHAGKQDFREDQDAAFFK    60
Baboon          MASGRARCTRKLRNWVVEQVESGQFPGVCWDDTAKTMFRIPWKHAGKQDFREDQDAAFFK    60
Green monkey    MASGRARCTRKLRNWVVEQVESGQFPGVCWDDTAKTMFRIPWKHAGKQDFREDQDAAFFK    60
                ************************************************************

Chimpanzee      AWAIFKGKYKEGDTGGPAVWKTRLRCALNKSSEFKEVPERGRMDVAEPYKVYQLLPPGTV    120
Orangutan       AWAIFKGKYKEGDTGGPAVWKTRLRCALNKSSEFKEVPERGRMDVAEPYKVYQLLPPGTV    120
Bonobo          AWAIFKGKYKEGDTGGPAVWKTRLRCALNKSSEFKEVPERGRMDVAEPYKVYQLLPPGTV    120
Human           AWAIFKGKYKEGDTGGPAVWKTRLRCALNKSSEFKEVPERGRMDVAEPYKVYQLLPPGIV    120
Leaf monkey     AWAIFKGKYKEGDTGGPAVWKTRLRCALNKSTEFEEVPKRGRMDVAEPYKVYRLLPPGPF    120
Macaque         AWAIFKGKYKEGDTGGPAVWKTRLRCALNKSTEFEEVPKRGCMDVAEPYKVYRLLPPGTF    120
Baboon          AWAIFKGKYKEGDTGGPAVWKTRLRCALNKSPEFEEVPKRGRMDVAEPYKVYRLLPPGTF    120
Green monkey    AWAIFKGKYKEGDTGGPAVWKTRLRCALNKSPEFEEVPKRGRMDVAEPYKVYRLLPPGTF    120
                ******************************* **:***:** **********:***** .

Chimpanzee      SGQPGTQKSPSKRHHSSVSSEREEEEDAMQNCTLSPSVLQDSLNNEEEGASGGAVHSDIG    180
Orangutan       SGQPGTQKSPSKRHHSSVSSEREEEEDAMQNCTLSPSVLQDSLNNEEEGASGGAVHSDIG    180
Bonobo          SGQPGTQKSPSKRHHSSVSSEREEEEDAMQNCTLSPSVLQDSLNNEEEGASGGAVHSDIG    180
Human           SGQPGTQKVPSKRQHSSVSSERKEEEDAMQNCTLSPSVLQDSLNNEEEGASGGAVHSDIG    180
Leaf monkey     SGQPGTQKSPSKRQHSSVSSERKEEAGAIQNCTLSPSVLQDSLNNEEEGASGGAVHSDIG    180
Macaque         SGQPGTQKSPSKRQHSSVSSERKEEEGARQNCTLSPSVLQESLNNEEEEASGGALHSDIG    180
Baboon          SGQPGTQKSPSKQQHSSVSSERKEEEGAMQNCTLSPSVLQESLNNEEEEASGGALHSDIG    180
Green monkey    SGQPGTQKSPSKRQHSSVSSERKEEEGAMQNCTLSPSVLQESLNNEEEEASGGALHSDIG    180
                ******** ***::********:** .* ***********:******* *****:*****

Chimpanzee      SSS-SSSPEPQEVTDTTEAPFQGDQRSLEFLLPPEPAPGSAVLSPDYSLLLTFIYNGRVV    239
Orangutan       SSS-SSSPEPQEVTDTTEAPFQGDQRSLEFLLPPEPAPGSAVLSPDYSLLLTFIYNGRVV    239
Bonobo          SSS-SSSPEPQEVTDTTEAPFQGDQRSLEFLLPPEPAPGSAVLSPDYSLLLTFIYNGRVV    239
Human           SSSSSSSPEPQEVTDTTEAPFQGDQRSLEFLLPPEPAPGRAVLSPDYSLLLTFIYNGRVV    240
Leaf monkey     SSS-SSSPEPQEGTDTTEAPFQGDQRSLEFLLPPE---------SDYSLLLTFIYNGRVV    230
Macaque         SSS-SSSPEPQEGTDTTEAPFQGDQRSLEFLLPPE---------PDYSLLLTFIYNGRVV    230
Baboon          SSS-SSSPEPQEGTDTTEAPFQGDQRSLEFLLPPE---------PDYSLLLTFIYNGRVV    230
Green monkey    SSS-SSSPESQEGTDTIEAPFQGDQRSLEFLLPPE---------PDYSLLLTFIYNGRVV    230
                *** ***** ** *** ******************          ***************
```

```
Chimpanzee      GEAQVQSLDCRLVAEPSGSESGMEQVLFPKPGPLEPTQRLLSQLERGILVASNPRGLFVQ    299
Orangutan       GEAQVQSLDCRLVAEPSGSESGMEQVLFPKPGPLEPTQRLLSQLERGILVASNPRGLFVQ    299
Bonobo          GEAQVQSLDCRLVAEPSGSESGMEQVLFPKPGPLEPTQRLLSQLERGILVASNPRGLFVQ    299
Human           GEAQVQSLDCRLVAEPSGSESSMEQVLFPKPGPLEPTQRLLSQLERGILVASNPRGLFVQ    300
Leaf monkey     GEAQVQSLDCRLVAEPSGSESSMEQVLFPKPGPLEPTQRLLSQLERGILVASNPRGLFVQ    290
Macaque         GEAQVQSLDCRLVAEPSGSESSMEQVLFPKPGPLEPTQRLLSQLERGILVASNPRGLFVQ    290
Baboon          GEAQVQSLDCRLVAEPSGSESSMEQVLFPKPGPLEPTQRLLSQLERGILVASNPRGLFVQ    290
Green monkey    GEAQVQSLDCRLVAEPSGSESSMEQVLFPKPGPLEPTQRLLSQLERGILVASNPRGLFVQ    290
                *********************.**************************************

Chimpanzee      RLCPIPISWNAPQAPPGPGPHLLPSNECVELFRTAYFCRDPPCSLCMALLRLCVAYPVWP    359
Orangutan       RLCPIPISWNAPQAPPGPGPHLLPSNECVELFRTAYFCRDPPCSLCMALLRLCVAYPVWP    359
Bonobo          RLCPIPISWNAPQAPPGPGPHLLPSNECVELFRTAYFCRDPPCSLCMALLRLCVAYPVWP    359
Human           RLCPIPISWNAPQAPPGPGPHLLPSNECVELFRTAYFCRDPPCSLCMALLRLCVAYPVWP    360
Leaf monkey     RLCPIPISWNAPQAPPGPGPHLLPSNECVELFRTAYFRRDPPCSLCMALLHLCGAHPVWP    350
Macaque         RLCPIPISWNAPQAPPGPGPHLLPSNECVELFRTAYFCRDPPCSLCMALLHLCGAHPVWS    350
Baboon          RLCPIPISWNAPQAPPGPGPHLLPSNECVELFRTAYFCRDPPCSLCMALLHLCGAHPVWP    350
Green monkey    RLCPIPISWNAPQAPPGPGPHLLPSNECVELFRTAYFCRDPPCSLCMALLHLCGAHPVWP    350
                ************************************* ************:** *:*** 

Chimpanzee      YAQPGSYLAALLQPRQWKRWFRLGQVLSGPGPPTEVPGNTEFLGREPWLQPYSTESYHSE    419
Orangutan       YAQPGSYLAALLQPRQWKRWFRLGQVLSGPGPPTEVPGNTEFLGREPWLQPYSTESYHSE    419
Bonobo          YAQPGSYLAALLQPRQWKRWFRLGQVLSGPGPPTEVPGNTEFLGREPWLQPYSTESYHSE    419
Human           YAQPGSYLAALLQPRQWKRWFRLGQVLSGPGPPTEVPGNTEFLGREPWLQPYSTESYHSE    420
Leaf monkey     YAQPGSYLAALLQPRQWKRWFRLGQVLPGPRPPTEVPGNTEFLGREPWPQPYSTESYHSE    410
Macaque         YAQPGSYLAALLQPRQWKRWFRLGQVLPGPRPPTEVPSNTEFLGREPWPQPYSTESYHSE    410
Baboon          YAQPGSYLAAILQPRQWKRWFR--------------------------------------    372
Green monkey    YAQPGSYLAALLQPRQWKRWFRLGQVLPGPRPPTEVPSNTEFLGREPWPQPYSTESYHSE    410
                **********:***********

Chimpanzee      DGAGLCPILAGADSRAAGSHSVPGVEPGGPIFHLTSLFFLSPLK      463
Orangutan       DGAGLCPILAGADSRAAGSHSVPGVEPGGPIFHLTSLFFLSPLK      463
Bonobo          DGAGLCPILAGADSRAAGSHSVPGVEPGGPIFHLTSLFFLSPLK      463
Human           DGAGLCPILAGADSRAAGSHSVPGVEPGGPIFHLTSLFFLSPLK      464
Leaf monkey     DGAGLCPILAGGDSRAAGSHSVPAVERGVPIFHLTSLFFLSPLK      454
Macaque         DGAGLCPILAGGDSRAAGSHSVPGVERGVPIFHLTSLFFLSPLK      454
Baboon          --------------------------------------------      372
Green monkey    DGAGLCPILAGGDSRAAGSHSVPGVERGVPIFHLTSLFFLSPLK      454
```

Figure S4

```
hIRF9A       MASGRARCTRKLRNWVVEQVESGQFPGVCWDDTAKTMFRIPWKHAGKQDFREDQDAAFFK     60
ColobusX1    MASGRARCTRKLRNWVVEQVESGQFPGVCWDDTAKTMFRIPWKHAGKQDFREDQDAAFFK     60
GorillaX2    MASGRARCTRKLRNWVVEQVESGQFPGVCWDDTAKTMFRIPWKHAGKQDFREDQDAAFFK     60
GorillaX1    MASGRARCTRKLRNWVVEQVESGQFPGVCWDDTAKTMFRIPWKHAGKQDFREDQDAAFFK     60
             ************************************************************

hIRF9A       AWAIFKGKYKEGDTGGPAVWKTRLRCALNKSSEFKEVPERGRMDVAEPYKVYQLLPPGIV     120
ColobusX1    AWAIFKGKYKEGDTGGPAVWKTRLRCALNKSPEFEEVPKRGRMDVAEPYKVYRLLPPGTF     120
GorillaX2    AWAIFKGKYKEGDTGGPAVWKTRLRCALNKSSEFKEVPERGRMDVAEPYKVYQLLPPGTV     120
GorillaX1    AWAIFKGKYKEGDTGGPAVWKTRLRCALNKSSEFKEVPERGRMDVAEPYKVYQLLPPGTV     120
             ******************************* **:***:*************:***** .

hIRF9A       SGQPGTQKVPSKRQHSSVSSERKEEEDAMQNCTLSPSVLQDSLNNEEEGASGGAVHSDIG     180
ColobusX1    SGQPGTQKSPSKRQHSSVSSERKEEAGAIQNCTLSPSVLQDSLNNEEEGASGGAVHSDIG     180
GorillaX2    SGQPGTQKSPSKRQHSSVSSERKEEEDAMQNCTLSPSVLQDSLNNEEEGASGGAVHSDIG     180
GorillaX1    SGQPGTQKSPSKRQHSSVSSERKEEEDAMQNCTLSPSVLQDSLNNEEEGASGGAVHSDIG     180
             ******** **************** .*:*******************************

hIRF9A       SSSSSSSPEPQEVTDTTEAPFQGDQRSLEFLLPPEPAPGRAVLSPDYSLLLTFIYNGRVV     240
ColobusX1    SSSSSSSPEPQEGTDTTEAPFQGDQRSLEFLLPPE---------SDYSLLLTFIYNGRVV     231
GorillaX2    SSS-SSSPEPQEVTDTTEAPFQGDQRSLEFLLPPE---------PDYSLLLTFIYNGRVV     230
GorillaX1    SSS-SSSPEPQEVTDTTEAPFQGDQRSLEFLLPPEPAPGRAVLSPDYSLLLTFIYNGRVV     239
             *** ******** **********************          ***************

hIRF9A       GEAQVQSLDCRLVAEPSGSESSMEQVLFPKPGPLEPTQRLLSQLERGILVASNPRGLFVQ     300
ColobusX1    GEAQVQSLDCRLVAEPSGSESSMEQVLFPKPGPLEPTQRLLSQLERGILVASNPRGLFVQ     291
GorillaX2    GEAQVQSLDCRLVAEPSGSESSMEQVLFPKPGPLEPTQRLLSQLERGILVASNPRGLFVQ     290
GorillaX1    GEAQVQSLDCRLVAEPSGSESSMEQVLFPKPGPLEPTQRLLSQLERGILVASNPRGLFVQ     299
             ************************************************************

hIRF9A       RLCPIPISWNAPQAPPGPGPHLLPSNECVELFRTAYFCRDPPCSLCMALLRLCVAYPVWP     360
ColobusX1    RLCPIPISWNAPQAPPGPGPHLLPSNECVELFRTAYFCRGEAALSGHLSSHPLPLSTPWA     351
GorillaX2    RLCPIPISWNAPQAPPGPGPHLLPSNECVELFRTAYFCRGEAVLSGHLSSHLLPLSTPWA     350
GorillaX1    RLCPIPISWNAPQAPPGPGPHLLPSNECVELFRTAYFCRGEAVLSGHLSSHLLPLSTPWA     359
             ***************************************.          :       * 

hIRF9A       YAQPGSYLAA----LLQPRQWKRWFRLGQVLSGPGPPTE-------------VPGNTEF     402
ColobusX1    QLGGSCHLPICQQILHVAYAWHFCTCVVHILCGHMPSLAATWQLSSSQDNGRGSSGRAQH     411
GorillaX2    QLVGSCYLPVCQQILHVAYAWHSCACVLHILCGHMPSLAATWQLSSSQDNRRGGSGRAQH     410
GorillaX1    QLVGSCYLPVCQQILHVAYAWHSCACVLHILCGHMPSLAATWQLSSSQDNRRGGSGRAQH     419
                 ..:*      *     *:    : ::*.*  *                   *.::.

hIRF9A       LGREPWLQPYSTESYHSEDGAGLCPILAGADSRAAGSHSVPGVEPGGPIFHLTSLFFLSP     462
ColobusX1    KSVVS-------------------------------------------------------     416
GorillaX2    KSVISWRTPSH-------------------------------------------------     421
GorillaX1    KSVISWRTPSH-------------------------------------------------     430
              .

hIRF9A       LK      464
ColobusX1    --      416
GorillaX2    --      421
GorillaX1    --      430
```

Figure S5

Supplemental Figure S5: Alignment of human PS-IRF9A sequences with mustelidae family members.

```
human       MASGRARCTRKLRNWVVEQVESGQFPGVCWDDTAKTMFRIPWKHAGKQDFREDQDAAFFK        60
otter       MASGRARCTRKLRNWVVEQVESGQFPGVCWEDAAKTMFRIPWKHAGKQDFREDQDAAFFK        60
ermine      MASGRARCTRKLRNWVVEQVESGQFPGVCWEDAAKTMFRIPWKHAGKQDFREDQDAAFFK        60
ferret      MASGRARCTRKLRNWVVEQVESGQFPGVCWEDAAKTMFRIPWKHAGKQDFREDQDAAFFK        60
            ******************************:*:***************************

human       AWAIFKGKYKEGDTGGPAVWKTRLRCALNKSSEFKEVPERGRMDVAEPYKVYQLLPP-GI        119
otter       AWAMFKGKYKEGDTEGPAIWKTRLRCALNKSSEFEEVPESGHRDGAEPYKVYRLLPPSGT        120
ermine      AWAMFKGKYKEGDTEGPAIWKTRLRCALNKSPEFEEVPESGHRDGAEPYKVYRLLPPSGT        120
ferret      AWAMFKGKYKEGDTEGPAIWKTRLRCALNKSPEFEEVPESGHRDGAEPYKVYRLLPPSGT        120
            ***:********** ***:************ **:**** *: * *******:**** *

human       VSGQPGTQKVPSKRQHSSVSSERKEEEDAMQNCTLSPSVLQDSLNNEEEGASGGAVHSDI        179
otter       CPAQPGTQKSPSKRSHSSVSSEKEEEEGTTKNCMLSPSWLEDPLRNEEVGANGGTGHSNF        180
ermine      CPAQPGTQKSPSKRCHSSVSSEKEEEECTTKNCMLSPSWLEDPLRNEEVGANGGTGHSNF        180
ferret      CSAQPGTQKSPSKRCHSFVSSEKEEEECTTKNCMLSPSWLEDPLRNEEVGANGGTGHSNF        180
              .****** **** ** ****::*** : :** **** *:* *.*** **.**: **::

human       GSSSS-SSSPEPQEVTDT-TEAPFQGDQRSLEFLLPPEPAPGRAVLSPDYSLLLTFIYNG        237
otter       GSSSSSSNSPEPQEGADTTEGAPFQGDQVSLEFLPPPD---------SDYSLLLTFIYGG        231
ermine      GSSSSSSNSPEPQEGADTTEGAPFQGDQVSLEFLPPPD---------SDYSLLLTFIYGG        231
ferret      GSSSSSSNSPEPQEGADTTEGAPFQGDQVSLEFLPPPD---------SDYSLLLTFIYGG        231
            ***** *.****** :**   ******* ***** **:          **********.*

human       RVVGEAQVQSLDCRLVAEPSGSESSMEQVLFPKPGPLEPTQRLLSQLERGILVASNPRGL        297
otter       RVVGEAQVQSLDCRLVAEPSGSQCGMEQVVFPKPDPREPTQRLLSQIERGVLVASNSRGL        291
ermine      RVVGEAQVQSLDCRLVAEPSGSQCGMEQVVFPKPDPREPTQRLLSQIERGVLVASNSRGL        291
ferret      RVVGEAQVQSLDCRLVAEPSGSQCGMEQVVFPKPDPREPTQRLLSQIERGVLVASNSRGL        291
            **********************:..****:****.* *********:***:***** ***

human       FVQRLCPIPISWNAPQAPPGPGPHLLPSNECVELFRTAYFCRDPPCSLCMALLRLCVAYP        357
otter       FVQRLCPIPVSWNAPQAPPGPGPHLLPSNECVELFRTTYFCRD-----------------        334
ermine      FVQRLCPIPVSWNAPQAPPGPGPHLLPSNECVELFRTTYFCRD-----------------        334
ferret      FVQRLCPIPVSWNAPQAPPGPGPHLLPSNECVELFRTTYFCRD-----------------        334
            *********:***************************:*****

human       VWPYAQPGSYLAALLQPRQWKRWFRLGQVLSGPGPPTEVPGNTEFLGREPWLQPYSTESY        417
otter       ------------------------------------------------------------        334
ermine      ------------------------------------------------------------        334
ferret      ------------------------------------------------------------        334

human       HSEDGAGLCPILAGADSRAAGSHSVPGVEPGGPIFHLTSLFFLSPLK-------      464
otter       ----GAGLCPTFAGGDSRGTGSLSVPAAEPGGPTFLLASLFLLSPLKETHSPHR      384
ermine      ----GAGLCPTFAGGDSRGTGSLSVPAAEPGGPTFLLAPLFLLSPLKETHSPHR      384
ferret      ----GAGLCPTFAGGDSRGTGSLSVPAAEPGGPTFLLAPLFLLSPLKETHSPHC      384
                ****** :**.***.:** ***..***** * *: **:*****
```

Table S1. Mammlian potein sequences used in this study. [a]

			IRF4		IRF8		IRF9		
**Species**	**Common name**	**Order (subgroup)**	**Accession #**	**Description**	**Accession #**	**Description**	**Accession #**	**Description**	**Motif**[b]
**[Eutherians]**									
Homo sapiens	**Human**	**Primate (great apes)**	NP_002451.2	interferon regulatory factor 4 isoform 1	NP_001350836.1	interferon regulatory factor 8 isoform 1	NP_006075.3	interferon regulatory factor 9 isoform 2	MEQAFAR
Pan paniscus	Bonobo	Primate (great apes)	XP_034817237.1	interferon regulatory factor 4 isoform X1	XP_003820892.1	interferon regulatory factor 8	XP_008959478.1	interferon regulatory factor 9 isoform X4	MEQAFAR
Gorilla gorilla gorilla	Gorilla	Primate (great apes)	XP_004043229.1	interferon regulatory factor 4 isoform X1	XP_018868405.1	interferon regulatory factor 8	XP_004055032.1	interferon regulatory factor 9 isoform X4	MEQAFAR
Pongo abelii	Sumatran orangutan	Primate (great apes)	XP_024104878.1	interferon regulatory factor 4 isoform X1	PNJ29652.1	IRF8 isoform 1	XP_002824643.1	interferon regulatory factor 9	MEQAFAR
Nomascus leucogenys	Northern white-cheeked gibb	Primate (lesser apes)	XP_003272225.1	interferon regulatory factor 4 isoform X1	XP_003272555.1	interferon regulatory factor 8	XP_012353449.1	interferon regulatory factor 9	MEQAFAR
Rhinopithecus bieti	Black snub-nosed monkey	Primate (Old World)	XP_017707346.1	PREDICTED: interferon regulatory factor 4 isoform X1	XP_017716051.1	PREDICTED: interferon regulatory factor 8 (141aa extra at N-term removed)	XP_017745706.1	PREDICTED: interferon regulatory factor 9 isoform X2	MEQAFAR
Colobus angolensis palliatus	Angola colobus	Primate (Old World)	XP_011810520.1	PREDICTED: interferon regulatory factor 4 isoform X1	XP_011783432.1	PREDICTED: interferon regulatory factor 8	XP_011786162.1	PREDICTED: interferon regulatory factor 9 isoform X2	MEQAFAR
Trachypithecus francoisi	François' langur	Primate (Old World)	XP_033071916.1	interferon regulatory factor 4 isoform X1	XP_033075113.1	interferon regulatory factor 8 isoform X1 (50aa extra at N-term removed)	XP_033044341.1	interferon regulatory factor 9 isoform X3	MEQAFAR
Macaca mulatta	Rhesus macaque	Primate (Old World)	NP_001253199.1	interferon regulatory factor 4	NP_001252887.1	interferon regulatory factor 8	NP_001247598.1	interferon regulatory factor 9	MEQAFAR
Theropithecus gelada	Gelada	Primate (Old World)	XP_025238217.1	interferon regulatory factor 4 isoform X1 (69aa extra at N-term removed)	XP_025226203.1	interferon regulatory factor 8 isoform X1	XP_025248553.1	interferon regulatory factor 9	MEQAFAR
Mandrillus leucophaeus	Drill	Primate (Old World)			XP_011826993.1	PREDICTED: interferon regulatory factor 8	XP_011826375.1	PREDICTED: interferon regulatory factor 9	MEQAFAR
Chlorocebus sabaeus	Green monkey	Primate (Old World)	XP_007972074.1	interferon regulatory factor 4 isoform X1	XP_007992460.1	interferon regulatory factor 8 isoform X1	XP_007984484.2	interferon regulatory factor 9 isoform X2	MEQAFAR
Callithrix jacchus	Common marmoset	Primate (New World)	XP_035151044.1	interferon regulatory factor 4 isoform X1	XP_035137716.1	interferon regulatory factor 8 isoform X1 (40aa extra at N-term removed)	XP_035116084.1	LOW QUALITY PROTEIN: E3 ubiquitin-protein ligase RNF31 (1096aa extra at N-term removed)	MEQAFAR
Aotus nancymaae	Nancy Ma's night monkey	Primate (New World)	XP_012320300.1	interferon regulatory factor 4 isoform X1	XP_012321516.1	interferon regulatory factor 8	XP_012323033.2	interferon regulatory factor 9 isoform X2	MEQAFAR
Cebus imitator	Panmanian white-faced capu	Primate (New World)	XP_017372729.1	interferon regulatory factor 4 isoform X1	XP_017399153.1	interferon regulatory factor 8 isoform X1 (58aa extra at N-term removed)	XP_017400192.1	interferon regulatory factor 9 isoform X2	MEQAFAR
Saimiri boliviensis boliviensis	Squirrel monkey	Primate (New World)	XP_039318619.1	interferon regulatory factor 4 isoform X1	XP_010331989.1	interferon regulatory factor 8	XP_010333313.2	interferon regulatory factor 9 isoform X2	MEQAFAR
Carlito syrichta	Philippine tarsier	Primate (prosimians)			XP_008061999.1	interferon regulatory factor 8	XP_008067926.1	interferon regulatory factor 9	MEQAFAR
Propithecus coquereli	Coquerel's sifaka	Primate (prosimians)	XP_012515575.1	PREDICTED: interferon regulatory factor 4 isoform X1	XP_012516717.1	PREDICTED: interferon regulatory factor 8	XP_012502733.1	PREDICTED: interferon regulatory factor 9 isoform X1	MEQAFAR
Lemur catta	Ring-tailed lemur	Primate (prosimians)	XP_045407514.1	interferon regulatory factor 4 isoform X2	XP_045389209.1	interferon regulatory factor 8	XP_045425895.1	interferon regulatory factor 9	MEQAFAR
Otolemur garnettii	Northern greater galago	Primate (prosimians)	XP_003799389.1	interferon regulatory factor 4 isoform X2	XP_003800834.1	interferon regulatory factor 8	XP_003801999.1	interferon regulatory factor 9	MEQAFAR
Galeopterus variegatus	Flying lemur	Dermoptera	XP_008581076.1	PREDICTED: interferon regulatory factor 4, partial (19 aa at N-term missing)	XP_008566160.1	PREDICTED: interferon regulatory factor 8	XP_008570555.1	PREDICTED: interferon regulatory factor 9	MEQAFAR
Tupaia chinensis	Chinese tree shrew	Scandentia	XP_006143117.1	interferon regulatory factor 4 isoform X1	ELW62919.1	Interferon regulatory factor 8	XP_006144618.1	interferon regulatory factor 9 isoform X3	MEQAFAR
Ictidomys tridecemlineatus	Ground squirrel	Rodentia	XP_021588817.1	interferon regulatory factor 4 isoform X1	KAG3256727.1	interferon regulatory factor 8	XP_013219933.1	interferon regulatory factor 9 isoform X3	MEQAFAR
Peromyscuc leucopus	White-footed mouse	Rodentia	XP_028736895.1	interferon regulatory factor 4 isoform X1	XP_028736640.1	interferon regulatory factor 8	XP_028747934.1	interferon regulatory factor 9 isoform X2	MEQAFAR
Mus musculus	House mouse	Rodentia	NP_038702.1	interferon regulatory factor 4 isoform a	NP_001288740.1	interferon regulatory factor 8	NP_032420.1	interferon regulatory factor 9 isoform 3	MEQAFAR
Rattus norvegicus	Brown rat	Rodentia	XP_006253961.1	interferon regulatory factor 4 isoform X1	NP_001008722.1	interferon regulatory factor 8	XP_006252036.1	interferon regulatory factor 9 isoform X2	MEQAFAR
Cavia porcellus	Guinea pig	Rodentia	XP_013000546.1	interferon regulatory factor 4 isoform X1	XP_003461072.1	LOW QUALITY PROTEIN: interferon regulatory factor 8	XP_013012890.1	interferon regulatory factor 9 isoform X2	MEQAFAR
Oryctolagus cuniculus	European rabbit	Lagomorpha	XP_002721677.1	PREDICTED: interferon regulatory factor 4 isoform X1	XP_002723728.1	PREDICTED: interferon regulatory factor 8	XP_002718143.1	PREDICTED: interferon regulatory factor 9 isoform X4	MEQAFAR
Ochotona curzoniae	Plateau pika	Lagomorpha	XP_040840044.1	interferon regulatory factor 4 isoform X1	XP_040838200.1	interferon regulatory factor 8 isoform X2	XP_040824320.1	interferon regulatory factor 9 isoform X5	MEQAFAR
Hyaena hyaena	Striped hyena	Carnivora (feliform)	XP_039078027.1	interferon regulatory factor 4	XP_039083681.1	interferon regulatory factor 8	XP_039110617.1	interferon regulatory factor 9 isoform X1	MEQAFAR
Felis catus	Cat	Carnivora (feliform)	XP_006931571.1	interferon regulatory factor 4 isoform X1	XP_023100909.1	interferon regulatory factor 8 isoform X1	XP_006932829.1	interferon regulatory factor 9 (38aa extra at N-term removed)	MEQAFAR
Leopardus geoffroyi	Geoffroy's cat	Carnivora (feliform)	XP_045353574.1	interferon regulatory factor 4 isoform X1	XP_045295861.1	interferon regulatory factor 8	XP_045306453.1	interferon regulatory factor 9 isoform X3	MEQAFAR
Canis lupus dingo	**Dingo**	**Carnivora (canid)**	XP_025302191.1	interferon regulatory factor 4 isoform X1	XP_025282824.1	interferon regulatory factor 8	XP_035575616.1	LOW QUALITY PROTEIN: interferon regulatory factor 9	MEQAFAR
Ursus arctos	Brown bear	Carnivora	XP_026367141.1	interferon regulatory factor 4 isoform X1	XP_026350439.1	interferon regulatory factor 8 isoform X2	XP_048069204.1	interferon regulatory factor 9 isoform X5	MEQAFAR
Mustela erminea	Short-tailed weasel	Carnivora (musteloid)	XP_032196818.1	interferon regulatory factor 4 isoform X1	XP_032179037.1	interferon regulatory factor 8 isoform X4	XP_032199788.1	interferon regulatory factor 9 isoform X2	MEQAFAR
Meles meles	European badger	Carnivora (musteloid)	XP_045860276.1	interferon regulatory factor 4 isoform X1	XP_045843460.1	interferon regulatory factor 8 isoform X2	XP_045863354.1	interferon regulatory factor 9	MEQAFAR
Odobenus rosmarus diverge	Pacific walrus	Carnivora (pinniped)	XP_004417493.1	interferon regulatory factor 4 isoform X1	XP_004392158.1	PREDICTED: interferon regulatory factor 8	XP_004402108.1	PREDICTED: interferon regulatory factor 9 isoform X2	MEQAFAR
Phoca vitulina	Harbor seal	Carnivora (pinniped)	XP_032280559.1	interferon regulatory factor 4-like (188aa extra at N-term removed)	XP_032245782.1	interferon regulatory factor 8	XP_032260698.1	interferon regulatory factor 9	MEQAFAR
Leptonychotes weddellii	Weddell seal	Carnivora (pinniped)	XP_006734466.1	interferon regulatory factor 4 isoform X1	XP_006743818.1	interferon regulatory factor 8	XP_006738726.1	interferon regulatory factor 9 isoform X2	MEQAFAR
Zalophus californianus	California sea lion	Carnivora (pinniped)	XP_027459737.1	interferon regulatory factor 4 isoform X1	XP_027474849.1	interferon regulatory factor 8	XP_027428451.1	interferon regulatory factor 9 isoform X1	MEQAFAR
Manis javanica	Sunda pangolin	Pholidota	XP_017536236.1	interferon regulatory factor 4 isoform X1	XP_036849697.1	interferon regulatory factor 8 isoform X1	XP_036740368.1	interferon regulatory factor 9	MEQAFAR
Camelus dromedarius	Arabian camel	Artiodactyla	XP_010974761.1	interferon regulatory factor 4 isoform X1	KAB1260723.1	Interferon regulatory factor 8 (11aa extra at N-term removed)	KAB1277093.1	Interferon regulatory factor 9	MEQAFAR
Muntiacus reevesi	Reeves's muntjac	Artiodactyla			KAB0382171.1	hypothetical protein FD755_004088	KAB0375395.1	hypothetical protein FD755_013887	MEQAFAR
Odocoileus virginianus texa	White-tailed deer	Artiodactyla	XP_020739876.1	interferon regulatory factor 4 isoform X1	XP_020755504.1	interferon regulatory factor 8 isoform X1 (12aa extra at N-term removed)	XP_020736608.1	interferon regulatory factor 9 isoform X2	MEQAFAR

			IRF4		IRF8		IRF9		
Species	Common name	Order (subgroup)	Accession #	Description	Accession #	Description	Accession #	Description	Motif[b]
Cervus elaphus	Red deer	Artiodactyla	XP_043764621.1	interferon regulatory factor 4 isoform X1	XP_043754158.1	interferon regulatory factor 8	XP_043775947.1	interferon regulatory factor 9	MEQAFAR
Bos taurus	**Cattle**	**Artiodactyla**	NP_001193091.1	interferon regulatory factor 4	XP_005218781.1	interferon regulatory factor 8 isoform X1	XP_010807205.1	interferon regulatory factor 9 isoform X2	MEQAFAR
Ovis aries	Sheep	Artiodactyla	XP_042092671.1	interferon regulatory factor 4 isoform X1	XP_027833509.1	interferon regulatory factor 8	XP_014952352.1	interferon regulatory factor 9 isoform X2	MEQAFAR
Sus scrofa	Pig	Artiodactyla	XP_020953623.1	interferon regulatory factor 4 isoform X1	NP_001239356.1	interferon regulatory factor 8	NP_001072138.1	1 interferon regulatory factor 9	MEQAFAR
Balaenoptera musculus	Blue whale	Artiodactyla (cetacea)			XP_036690945.1	interferon regulatory factor 8 isoform X4	XP_036697685.1	interferon regulatory factor 9	MEQAFAR
Balaenoptera acutorostrata	Minke whale	Artiodactyla (cetacea)	XP_007173942.1	interferon regulatory factor 4 isoform X1					MEQAFAR
Physeter catodon	Sperm whale	Artiodactyla (cetacea)	XP_007101199.1	interferon regulatory factor 4 isoform X1	XP_028334161.1	interferon regulatory factor 8 (38aa extra at N-term removed)	XP_007130119.1	interferon regulatory factor 9	MEQAFAR
Neophocaena asiaeorientali	Yangtze finless porpoise	Artiodactyla (cetacea)	XP_024622173.1	interferon regulatory factor 4	XP_024591468.1	interferon regulatory factor 8 isoform X2	XP_024608961.1	interferon regulatory factor 9	MEQAFAR
Orcinus orca	Orca	Artiodactyla (cetacea)	XP_004281126.1	interferon regulatory factor 4 isoform X1	XP_004280125.1	interferon regulatory factor 8 isoform X4	XP_004283186.1	interferon regulatory factor 9 isoform X1	MEQAFAR
Equus przewalskii	Przewalski's horse	Perissodactyla	XP_008526673.1	PREDICTED: interferon regulatory factor 4 isoform X1	XP_008514974.1	PREDICTED: interferon regulatory factor 8 isoform X1	XP_008537764.1	interferon regulatory factor 9 isoform X1	MEQAFAR
Ceratotherium simum simur	White rhinoceros	Perissodactyla	XP_004419242.1	PREDICTED: interferon regulatory factor 4 isoform X1	XP_014648136.1	PREDICTED: interferon regulatory factor 8	XP_014635947.1	PREDICTED: interferon regulatory factor 9	MEQAFAR
Pteropus alecto	**Black flying fox**	**Chiroptera (Yinptero)**	XP_006918921.1	interferon regulatory factor 4 isoform X1	XP_006926799.1	interferon regulatory factor 8 isoform X2	XP_006913612.1	interferon regulatory factor 9 isoform X1	MEQAFAR
Rousettus aegyptiacus	Egyptian fruit bat	Chiroptera (Yinptero)	XP_016014999.1	interferon regulatory factor 4 isoform X1	XP_015984966.2	interferon regulatory factor 8 isoform X2	XP_015976035.2	interferon regulatory factor 9	MEQAFAR
Rhinolophus sinicus	Chinese rufous horseshoe ba	Chiroptera (Yinptero)	XP_019570449.1	PREDICTED: interferon regulatory factor 4 isoform X1	XP_019602691.1	interferon regulatory factor 8 isoform X2	XP_019569947.1	PREDICTED: LOW QUALITY PROTEIN: interferon regulatory factor 9	MEQAFAR
Hipposideros armiger	Great roundleaf bat	Chiroptera (Yinptero)	XP_019484966.1	PREDICTED: interferon regulatory factor 4, partial (67aa at N-term missing)	XP_019490770.1	PREDICTED: interferon regulatory factor 8	XP_019518716.1	PREDICTED: interferon regulatory factor 9	MEQAFAR
Myotis myotis	Greater mouse-eared bat	Chiroptera (Yango)	XP_036207524.1	interferon regulatory factor 4 isoform X2	XP_036202256.1	interferon regulatory factor 8 isoform X2	KAF6387082.1	interferon regulatory factor 9	MEQAFAR
Molossus molossus	Velvety free-tailed bat	Chiroptera (Yango)	XP_036112174.1	interferon regulatory factor 4 isoform X1	XP_036131548.1	interferon regulatory factor 8	XP_036119340.1	interferon regulatory factor 9 isoform X1	MEQAFAR
Miniopterus natalensis	Natal long-fingered bat	Chiroptera (Yango)	XP_016061862.1	PREDICTED: interferon regulatory factor 4 isoform X1	XP_016053440.1	PREDICTED: interferon regulatory factor 8	XP_016061354.1	PREDICTED: interferon regulatory factor 9 isoform X1	MEQAFAR
Phyllostomus discolor	Pale spear-nosed bat	Chiroptera (Yango)	XP_028369976.1	interferon regulatory factor 4 isoform X1	XP_028357964.1	interferon regulatory factor 8	KAF6130511.1	interferon regulatory factor 9	MEQAFAR
Desmodus rotundus	Common vampire bat	Chiroptera (Yango)	XP_024408170.1	interferon regulatory factor 4 isoform X1	XP_024412009.1	interferon regulatory factor 8	XP_045047970.1	LOW QUALITY PROTEIN: interferon regulatory factor 9	MEQAFAR
Sturnira hondurensis	Honduran yellow-shouldered	Chiroptera (Yango)	XP_036926336.1	interferon regulatory factor 4 isoform X1	XP_036896139.1	interferon regulatory factor 8	XP_036893479.1	interferon regulatory factor 9 isoform X2	MEQAFAR
Artibeus jamaicensis	Jamaican fruit bat	Chiroptera (Yango)	XP_037012920.1	interferon regulatory factor 4 isoform X1	XP_036998280.1	interferon regulatory factor 8	XP_036985338.1	interferon regulatory factor 9	MEQAFAR
Loxodonta africana	African bush elephant	Afrotheria	XP_003417904.1	interferon regulatory factor 4 isoform X2	XP_003418123.2	interferon regulatory factor 8 (33aa extra at N-term removed)	XP_003421047.1	interferon regulatory factor 9 isoform X2	MEQAFAR
Talpa occidentalis	Spanish mole	Eulipotyphla	XP_037375057.1	interferon regulatory factor 4 isoform X1	XP_037371658.1	interferon regulatory factor 8	XP_037357715.1	interferon regulatory factor 9	MEQAFAR
Erinaceus europaeus	European hedgehog	Eulipotyphla	XP_007531420.1	PREDICTED: interferon regulatory factor 4 isoform X1	XP_016047237.1	PREDICTED: interferon regulatory factor 8 isoform X1	XP_016049838.1	PREDICTED: LOW QUALITY PROTEIN: interferon regulatory factor 9	MEQAFAR
Choloepus didactylus	**Linnaeus's two-toed sloth**	**Xenarthra**	XP_037700109.1	interferon regulatory factor 4 isoform X1	XP_037671802.1	interferon regulatory factor 8 isoform X2	XP_037690555.1	interferon regulatory factor 9	MEQAFAR
**[Marsupials]**									
Monodelphis domestica	Gray short-tailed opossum	Didelphimorphia	XP_001378726.2	PREDICTED: interferon regulatory factor 4	XP_001365777.1	PREDICTED: interferon regulatory factor 8 isoform X3	XP_007479923.1	PREDICTED: interferon regulatory factor 9 isoform X4	MEQAFAR
Gracilinanus agilis	Agile gracile opossum	Didelphimorphia	XP_044539710.1	interferon regulatory factor 4 (45aa at N-term not alignable and excluded)	XP_044516480.1	interferon regulatory factor 8 isoform X1	XP_044521155.1	interferon regulatory factor 9 isoform X2	MEQAFAR
Dromiciops gliroides	Colocolo opossum	Microbiotheria	XP_043834132.1	interferon regulatory factor 4	XP_043842845.1	interferon regulatory factor 8	XP_043835967.1	interferon regulatory factor 9 isoform X3	MEQAFAR
Vombatus ursinus	**Common wombat**	**Diprotodontia**	XP_027697513.1	interferon regulatory factor 4 isoform X1	XP_027703450.1	interferon regulatory factor 8	XP_027698833.1	interferon regulatory factor 9 isoform X5	MEQAFAR
Phascolarctos cinereus	Koala	Diprotodontia	XP_020832338.1	interferon regulatory factor 4	XP_020844889.1	interferon regulatory factor 8 isoform X2	XP_020823706.1	interferon regulatory factor 9 isoform X4	MEQAFAR
Trichosurus vulpecula	Common brushtail possum	Diprotodontia	XP_036597284.1	interferon regulatory factor 4	XP_036604271.1	interferon regulatory factor 8	XP_036592021.1	interferon regulatory factor 9 isoform X2	MEQAFAA
Sarcophilus harrisii	Tasmanian devil	Dasyuromorphia	XP_003760275.2	interferon regulatory factor 4	XP_003758515.1	interferon regulatory factor 8	XP_023355306.1	interferon regulatory factor 9 isoform X2	MEQAFVR
**[Monotremes]**									
Tachyglossus aculeatus	Soft-beaked echidna	Monotremata	XP_038624022.1	interferon regulatory factor 4	XP_038610420.1	interferon regulatory factor 8	XP_038597164.1	interferon regulatory factor 9	LEQIFAR
Ornithorhynchus anatinus	**Platypus**	**Monotremata**	XP_028909458.1	interferon regulatory factor 4	XP_028931568.1	interferon regulatory factor 8	XP_028934200.1	interferon regulatory factor 9	LEQTFAR

[a]IRF family classification is based on the phylogenetic placement. Species shown in boldface fonts are included in the phylogeny shown in Figure 2. Sequences with ambiguous or incorrect annotations are shown with yellow background.

[b]Positions different from the motif "MEQAFAR" are shown in red.

Table S2. IRF4/8/9 potein sequences from other vertebrates used in this study.[a]

			IRF4		IRF8		IRF9		
**Species**	**Common name**	**Order (subgroup)**	**Accession #**	**Description**	**Accession #**	**Description**	**Accession #**	**Description**	**Motif[b]**
**[Cartilaginous fish]**									
Callorhinchus milii	Australian ghostshark	Chimaeriformes	XP_007887807.1	interferon regulatory factor 4a isoform X2	XP_007887425.1	interferon regulatory factor 8			
Chiloscyllium plagiosum	Whitespotted bamboo shark	Orectolobiformes	XP_043575316.1	interferon regulatory factor 4-like isoform X3	XP_043562552.1	interferon regulatory factor 8 isoform X2	XP_043538238.1	interferon regulatory factor 8-like	VYQVAAK
Carcharodon carcharias	Great white shark	Lamniformes	XP_041040959.1	interferon regulatory factor 4a	XP_041047124.1	interferon regulatory factor 8	XP_041036749.1	interferon regulatory factor 8-like isoform X2	IYQMAAK
**[Ray-finned fish]**									
Polypterus senegalus	Senegal bichir	Polypteriformes	XP_039609366.1	interferon regulatory factor 4a	XP_039619327.1	interferon regulatory factor 8 isoform X1	XP_039603616.1	interferon regulatory factor 9	VEQPWAR
Danio rerio	Zebrafish	Cypriniformes	NP_001116182.1	interferon regulatory factor 4a	NP_001002622.1	interferon regulatory factor 8	NP_991273.2	interferon regulatory factor 9	ISLPWAE
**[Lobe-finned fish]**									
Protopterus annectens	West African lungfish	Dipnoi	XP_043921088.1	interferon regulatory factor 4	XP_043937695.1	interferon regulatory factor 8 isoform X1	XP_043914370.1	interferon regulatory factor 8-like	IEQLFAG
Latimeria chalumnae	West Indian Ocean coelacanth	Coelacanthiformes	XP_005995158.1	PREDICTED: interferon regulatory factor 4	XP_006010434.1	PREDICTED: interferon regulatory factor 8	XP_006010095.1	PREDICTED: interferon regulatory factor 8-like	IEQEFAR
**[Amphibians]**									
Microcaecilia unicolor	caecilian	Gymnophiona	XP_030045938.1	interferon regulatory factor 4	XP_030058565.1	LOW QUALITY PROTEIN: interferon regulatory factor 8	XP_030043228.1	interferon regulatory factor 9 isoform X1	VEQVSAK
Rhinatrema bivittatum	Two-lined caecilian	Gymnophiona	XP_029446387.1	interferon regulatory factor 4	XP_029463934.1	interferon regulatory factor 8 isoform X3	XP_029437842.1	interferon regulatory factor 9	IEQVMAK
Xenopus tropicalis	Western clawed frog	Anura	XP_002936464.1	interferon regulatory factor 4	XP_004913664.2	interferon regulatory factor 8	XP_002939071.3	interferon regulatory factor 9 isoform X1	IEQVMAS
Rana temporaria	Common frog	Anura	XP_040209569.1	interferon regulatory factor 4	XP_040185344.1	interferon regulatory factor 8	XP_040210590.1	interferon regulatory factor 9	IEQVMAS
Bufo bufo	Common toad	Anura	XP_040287899.1	interferon regulatory factor 4	XP_040266707.1	interferon regulatory factor 8	XP_040272853.1	interferon regulatory factor 9 isoform X1	IEQILAS
**[Squamata]**									
Varanus komodoensis	Komodo dragon	Squamata	XP_044280476.1	interferon regulatory factor 4	XP_044283862.1	interferon regulatory factor 8 isoform X2	XP_044276889.1	interferon regulatory factor 9 isoform X3	MEQILAH
Pogona vitticeps	Central bearded dragon	Squamata	XP_020645470.1	interferon regulatory factor 4	XP_020664143.1	interferon regulatory factor 8 isoform X1	XP_020666164.1	interferon regulatory factor 9	IEPVCAL
Anolis carolinensis	Green anole	Squamata	XP_003224441.2	PREDICTED: interferon regulatory factor 4 isoform X1	XP_003227167.2	PREDICTED: interferon regulatory factor 8	XP_008123187.1	PREDICTED: interferon regulatory factor 9	MEQINAV
Sceloporus undulatus	Eastern fence lizard	Squamata	XP_042320981.1	LOW QUALITY PROTEIN: interferon regulatory factor 4	XP_042294683.1	interferon regulatory factor 8 isoform X2	XP_042327877.1	interferon regulatory factor 9 isoform X3	MEQIIPF
Zootoca vivipara	Viviparous lizard	Squamata	XP_034982261.1	interferon regulatory factor 4 isoform X2	XP_034976470.1	interferon regulatory factor 8 isoform X2	XP_034992164.1	interferon regulatory factor 9	-
Lacerta agilis	Sand lizard	Squamata	XP_033010361.1	interferon regulatory factor 4 isoform X2	XP_033012809.1	interferon regulatory factor 8	XP_033026855.1	interferon regulatory factor 9	MEQIFAS
Podarcis muralis	Common wall lizard	Squamata	XP_028593681.1	interferon regulatory factor 4	[a]Positions differen	interferon regulatory factor 8	XP_028559336.1	interferon regulatory factor 9	MEQIFAS
Python bivittatus	Burmese python	Squamata	XP_015742815.2	interferon regulatory factor 4 isoform X2	XP_015743398.1	interferon regulatory factor 8	XP_015744954.1	interferon regulatory factor 9	MEQAFAL
Crotalus tigris	Tiger rattlesnake	Squamata	XP_039203094.1	interferon regulatory factor 4 isoform X2	XP_039211976.1	interferon regulatory factor 8	XP_039194000.1	interferon regulatory factor 9 isoform X2	MEQAFTI
Pseudonaja textilis	Eastern brown snake	Squamata	XP_026570818.1	interferon regulatory factor 4	XP_026569774.1	interferon regulatory factor 8	XP_026575304.1	interferon regulatory factor 9	MEQTFAI
Sphaerodactylus townsendi	Townsend's dwarf sphaero	Squamata	XP_048364002.1	interferon regulatory factor 4	XP_048371833.1	interferon regulatory factor 8 isoform X1	XP_048373305.1	interferon regulatory factor 9 isoform X3	MAQNFAA
**[Turtles]**									
Mauremys mutica	Yellow pond turtle	Testudines	XP_044859100.1	LOW QUALITY PROTEIN: interferon regulatory factor 4 (133aa extra at N-term excluded)	XP_044843772.1	interferon regulatory factor 8 isoform X1	XP_044842515.1	interferon regulatory factor 9 isoform X2	MEQAFAR
Dermochelys coriacea	Leatherback sea turtle	Testudines	XP_038248972.1	interferon regulatory factor 4 isoform X4	XP_038224849.1	interferon regulatory factor 8 isoform X1	XP_038225070.1	interferon regulatory factor 9	MEQTFAR
Pelodiscus sinensis	Chinese softshell turtle	Testudines	XP_006138885.1	interferon regulatory factor 4	XP_006137424.1	interferon regulatory factor 8			
[Archosauriformes]									
Alligator mississippiensis	American alligator	Crocodilia	XP_006276607.1	PREDICTED: interferon regulatory factor 4	XP_006276758.1	PREDICTED: interferon regulatory factor 8 isoform X2	XP_019343804.1	PREDICTED: interferon regulatory factor 9 isoform X3	MEQAFAL
Gallus gallus	Red junglefowl	Galliformes	NP_989630.2	interferon regulatory factor 4	NP_990747.1	interferon regulatory factor 8	(NP_989889.1)[c]	(interferon regulatory factor 9)[c]	

[a]IRF family classification is based on the phylogenetic placement. Sequences with ambiguous or incorrect annotations are shown with yellow background.

[b]Positions different from the motif "MEQAFAR" are shown in red. In the case of the Z. vivipara protein (shown as '-'), just before the motif region, the sequence changes significantly. Correct exon may not be used (no other isoform is predicted).

[c]Although NP_989889.1 is annotated as IRF9, it clusters with IRF 4-like proteins on the phylogeny. Therefore, it was excluded from this study.

Table S3. IR6 protein sequences used as the outgroup in this study.

Species	Common name	Order (subgroup)	Accession #	Description
**[Eutherians]**				
Homo sapiens	Human	Primate (great apes)	NP_006138.1	interferon regulatory factor 6 isoform 1
**[Cartilaginous fish]**				
Callorhinchus milii	Australian ghostshark	Chimaeriformes	XP_007897748.1	interferon regulatory factor 6
Chiloscyllium plagiosum	Whitespotted bamboo shark	Orectolobiformes	XP_043572560.1	interferon regulatory factor 6
**[Ray-finned fish]**				
Polypterus senegalus	Senegal bichir	Polypteriformes	XP_039604520.1	interferon regulatory factor 6
Danio rerio	Zebrafish	Cypriniformes	NP_956892.1	interferon regulatory factor 6
**[Lobe-finned fish]**				
Protopterus annectens	West African lungfish	Dipnoi	XP_043933055.1	interferon regulatory factor 6 isoform X2
Latimeria chalumnae	West Indian Ocean coelacanth	Actinistia	XP_014347913.1	PREDICTED: interferon regulatory factor 6
**[Amphibians]**				
Xenopus tropicalis	Western clawed frog	Anura	NP_001025493.1	interferon regulatory factor 6
**[Archosauriformes]**				
Alligator mississippiensis	American alligator	Crocodilia	XP_006268141.1	PREDICTED: interferon regulatory factor 6 isoform X2
Gallus gallus	Red junglefowl	Galliformes	XP_015154525.1	interferon regulatory factor 6 isoform X4

Table S4. Primate IRF9 potein sequences used in this study

Accession Number	Full Name
NP_001372329.1	interferon regulatory factor 9 isoform 1 [Homo sapiens]
NP_001372330.1	interferon regulatory factor 9 isoform 3 [Homo sapiens]
NP_001372331.1	interferon regulatory factor 9 isoform 4 [Homo sapiens]
NP_006075.3	interferon regulatory factor 9 isoform 2 [Homo sapiens]
XP_003801999.1	interferon regulatory factor 9 [Otolemur garnettii]
XP_039324659.1	interferon regulatory factor 9 isoform X3 [Saimiri boliviensis boliviensis]
XP_010333313.2	interferon regulatory factor 9 isoform X2 [Saimiri boliviensis boliviensis]
XP_039324658.1	interferon regulatory factor 9 isoform X1 [Saimiri boliviensis boliviensis]
XP_007984484.2	interferon regulatory factor 9 isoform X2 [Chlorocebus sabaeus]
XP_007984483.2	interferon regulatory factor 9 isoform X1 [Chlorocebus sabaeus]
XP_037600275.1	interferon regulatory factor 9 isoform X1 [Cebus imitator]
XP_017400192.1	interferon regulatory factor 9 isoform X2 [Cebus imitator]
XP_034793221.1	interferon regulatory factor 9 isoform X3 [Pan paniscus]
XP_008959478.1	interferon regulatory factor 9 isoform X4 [Pan paniscus]
XP_008959477.1	interferon regulatory factor 9 isoform X2 [Pan paniscus]
XP_008959476.1	interferon regulatory factor 9 isoform X1 [Pan paniscus]
XP_003809121.1	interferon regulatory factor 9 isoform X5 [Pan paniscus]
XP_033044341.1	interferon regulatory factor 9 isoform X3 [Trachypithecus francoisi]
XP_033044340.1	interferon regulatory factor 9 isoform X2 [Trachypithecus francoisi]
XP_033044339.1	interferon regulatory factor 9 isoform X1 [Trachypithecus francoisi]
XP_032035985.1	interferon regulatory factor 9 [Hylobates moloch]
XP_032121534.1	interferon regulatory factor 9 [Sapajus apella]
XP_031791671.1	interferon regulatory factor 9 [Piliocolobus tephrosceles]
XP_021796767.1	interferon regulatory factor 9 isoform X5 [Papio anubis]
XP_021796766.1	interferon regulatory factor 9 isoform X4 [Papio anubis]
XP_021796765.1	interferon regulatory factor 9 isoform X3 [Papio anubis]
XP_021796764.1	interferon regulatory factor 9 isoform X2 [Papio anubis]
XP_021796763.1	interferon regulatory factor 9 isoform X1 [Papio anubis]
XP_018864870.1	interferon regulatory factor 9 isoform X3 [Gorilla gorilla gorilla]
XP_018864869.1	interferon regulatory factor 9 isoform X2 [Gorilla gorilla gorilla]
XP_018864868.1	interferon regulatory factor 9 isoform X1 [Gorilla gorilla gorilla]
XP_004055032.1	interferon regulatory factor 9 isoform X4 [Gorilla gorilla gorilla]
XP_010362078.1	interferon regulatory factor 9 [Rhinopithecus roxellana]
XP_012353449.1	interferon regulatory factor 9 [Nomascus leucogenys]
NP_001247598.1	interferon regulatory factor 9 [Macaca mulatta]
XP_014998835.2	interferon regulatory factor 9 isoform X1 [Macaca mulatta]
XP_025248553.1	interferon regulatory factor 9 [Theropithecus gelada]
XP_011732994.1	interferon regulatory factor 9 [Macaca nemestrina]
XP_001167256.3	interferon regulatory factor 9 isoform X4 [Pan troglodytes]
XP_009425840.3	interferon regulatory factor 9 isoform X3 [Pan troglodytes]
XP_009425839.3	interferon regulatory factor 9 isoform X2 [Pan troglodytes]

XP_009425838.3	interferon regulatory factor 9 isoform X1 [Pan troglodytes]
XP_021574105.1	interferon regulatory factor 9 [Carlito syrichta]
XP_021530555.1	interferon regulatory factor 9 isoform X3 [Aotus nancymaae]
XP_021530554.1	interferon regulatory factor 9 isoform X2 [Aotus nancymaae]
XP_021530553.1	interferon regulatory factor 9 isoform X1 [Aotus nancymaae]
XP_020141443.1	interferon regulatory factor 9 isoform X1 [Microcebus murinus]
XP_012611454.1	interferon regulatory factor 9 isoform X2 [Microcebus murinus]
XP_017745706.1	PREDICTED: interferon regulatory factor 9 isoform X2 [Rhinopithecus bieti]
XP_017745705.1	PREDICTED: interferon regulatory factor 9 isoform X1 [Rhinopithecus bieti]
XP_005560993.1	PREDICTED: interferon regulatory factor 9 isoform X1 [Macaca fascicularis]
XP_012502734.1	PREDICTED: interferon regulatory factor 9 isoform X2 [Propithecus coquereli]
XP_012502733.1	PREDICTED: interferon regulatory factor 9 isoform X1 [Propithecus coquereli]
XP_011946607.1	PREDICTED: interferon regulatory factor 9 [Cercocebus atys]
XP_011786162.1	PREDICTED: interferon regulatory factor 9 isoform X2 [Colobus angolensis palliatus]
XP_011786161.1	PREDICTED: interferon regulatory factor 9 isoform X1 [Colobus angolensis palliatus]
XP_011826375.1	PREDICTED: interferon regulatory factor 9 [Mandrillus leucophaeus]
JAB38301.1	interferon regulatory factor 9 [Callithrix jacchus]
PNJ35602.1	IRF9 isoform 3 [Pongo abelii]
PNJ35601.1	IRF9 isoform 1 [Pongo abelii]
PNI96744.1	IRF9 isoform 2 [Pan troglodytes]
XP_028934201.1	interferon regulatory factor 9 [Ornithorhynchus anatinus]
